# Bidirectional Communication Between Astrocytes and Neurons via Extracellular Vesicles: A Multi‐Omics Approach

**DOI:** 10.1111/jnc.70373

**Published:** 2026-02-06

**Authors:** Daria Hajka, Paulina Żebrowska‐Różańska, Katarzyna Romańczuk, Jacek R. Wiśniewski, Łukasz Łaczmański, Norbert Łodej, Krzysztof J. Pawlik, Dariusz Rakus, Agnieszka Gizak

**Affiliations:** ^1^ Department of Molecular Physiology and Neurobiology University of Wrocław Wrocław Poland; ^2^ Hirszfeld Institute of Immunology and Experimental Therapy, Polish Academy of Sciences Wrocław Poland; ^3^ Department of Proteomics and Signal Transduction Max Planck Institute of Biochemistry Martinsried Germany

**Keywords:** astrocytes, extracellular vesicles, neurons, proteomics, RNA‐seq

## Abstract

Cells modulate their physiology through multiple mechanisms—cell–cell contacts and autocrine/paracrine signaling, including via extracellular vesicles (EVs). In this study, we exposed mouse hippocampal astrocyte and neuron monocultures to EVs from the opposing cell type and subsequently performed RNA sequencing to examine transcriptomic changes. Mass spectrometry was used to analyze the proteomes of EVs from astrocyte and neuron monocultures, as well as from astrocyte‐neuron co‐cultures, to investigate the molecular basis of EVs‐induced transcriptomic alterations and to determine the extent to which cells adjust EV cargo in response to feedback signals. EVs secreted by both cell types induced cell‐specific transcriptomic changes in target cells, related to migration, proliferation, differentiation, and energy production. Unique changes in the proteome of EVs from astrocytic‐neuronal co‐cultures highlighted the dynamic regulation of signaling molecule secretion via cell interactions.

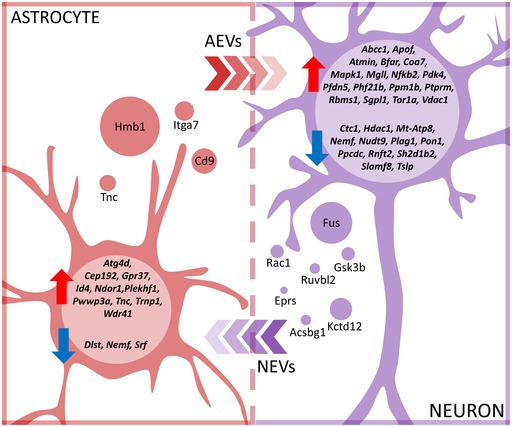

## Graphical abstract

Schematic diagram illustrating the communication between mouse hippocampal astrocytes and neurons through extracellular vesicles (EVs). The illustration highlights unique proteins secreted by each cell type within EVs, with vesicle size proportional to the concentration of secreted proteins. Additionally, within the cells, selected genes that are upregulated in response to EVs from the opposite cell type are marked with red arrows, while those downregulated are marked with blue arrows. AEVs, astrocytic extracellular vesicles; NEVs, neuronal extracellular vesicles.

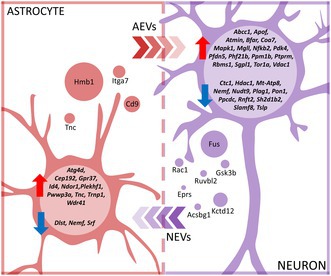

**Abbreviations**
A: untreated monoculture of astrocytes
AEVs: astrocytic extracellular vesicles
AN: monoculture of astrocytes exposed to neuronal EVs
Ara‐C: cytosine arabinoside
ATP: adenosine triphosphate
BH: Benjamini‐Hochberg
BP: biological process
COEVs: astrocyte‐neuron co‐culture extracellular vesicles
DEGs: Differentially Expressed Genes
DMEM: Dulbecco's Modified Eagle Medium
EVs: extracellular vesicles
FBS: fetal bovine serum
FDR: false discovery rate
GO: Gene Ontology
GSEA: Gene Set Enrichment Analysis
IQR: interquartile range
KEGG: Kyoto Encyclopedia of Genes and Genomes
LC–MS/MS: Liquid Chromatography—Tandem Mass Spectrometry
MED‐FASP: Multienzyme Digestion—Filter Aided Sample Preparation
MISEV: Minimal Information for Studies of Extracellular Vesicles
N: untreated monoculture of neurons
NA: monoculture of neurons exposed to astrocytic EVs
NEVs: neuronal extracellular vesicles
NGS: next‐generation sequencing
NMDAR: N‐Methyl‐D‐Aspartate Receptor
PBS: phosphate‐buffered saline
PCA: principal component analysis
RIN: RNA integrity number
RNA‐seq: RNA sequencing
ROS: reactive oxygen species
RRID: Research Resource Identifier (see scicrunch.org)
TCA: tricarboxylic acid cycle
TEM: transmission electron microscopy
TPA: total protein approach

## 1 Introduction

The intricate set of interactions between different cell types—called cell‐to‐cell cross‐talk—that influence their physiology and shape the environment can occur through multiple mechanisms, not only through the “classical” physical contacts between these cells. One such mechanism is the release of vesicles into the intercellular space. The so‐called extracellular vesicles (EVs) are a heterogeneous group of membrane‐bound structures that transport nucleic acids, lipids, proteins, and even organelles (Crewe et al. 2021) between cells. Their complex cargo composition reflects the physiological or pathological state of the originating cells (Lo Cicero et al. 2015). This unique feature makes EVs highly promising as biomarkers for a wide range of diseases, including neurodegenerative disorders, since changes in their cargo profiles can occur long before the first clinical symptoms appear (Rao et al. 2013).

In the brain, astrocytes and neurons cooperate at multiple levels to sustain neural function and overall brain homeostasis (Pellerin and Magistretti 2012). The long‐known interaction called astrocyte‐neuron cross‐talk takes place, among others, via EVs. EVs content and its impact on the physiology of both cell types have been presented in numerous publications (Almansa et al. 2022; Gosselin et al. 2013; Hajka et al. 2024; Pascua‐Maestro et al. 2019; Taylor et al. 2007; Wang et al. 2011), but our knowledge on this subject is still far from complete. Many of these studies have focused on EVs isolated from either astrocytic or neuronal monocultures. However, cell–cell interactions in co‐culture conditions—which more closely reflect the physiological situation in the brain—may reshape not only specific molecular pathways within the cells but also the cargo of secreted EVs.

In this study, we used LC–MS/MS to characterize the protein content of EVs secreted by monocultures of hippocampal astrocytes and neurons. To further explore the mutual influence of astrocytes and neurons on the vesicles they release, we also examined the proteome of EVs derived from astrocyte–neuron co‐cultures and compared it with the proteomes of EVs from monocultures.

Next‐Generation Sequencing (NGS) has revolutionized the ability to perform high‐resolution, comprehensive transcriptome analyses, offering unprecedented insights into gene expression dynamics and cellular responses to a variety of stimuli (Satam et al. 2023). Therefore, in parallel to proteomic study, we used RNA‐seq to gain some insight into the transcriptomic changes induced in mouse hippocampal astrocyte cultures by the cargo of neuron‐secreted vesicles and in neuronal cultures by the content of astrocyte vesicles. Moreover, based on literature data, we sought to determine whether specific proteins identified in EV cargo could be linked to the initiation of observed transcriptomic responses.

This integrative approach allowed us to explore not only how reciprocal EV‐mediated communication shapes cell‐type‐specific molecular landscapes, but also how interactions between neurons and astrocytes influence the molecular composition of the vesicles they release, thereby providing new insights into the dynamic bidirectional regulation of neuronal and astrocytic interactions.

## 2 Materials and Methods

### 2.1 Cell Culture and Treatment

Animals were sourced from the Experimental Animal Facility at the Medical University of Wrocław. A total of 340 mice were used in the study. Of these, 300 animals contributed to the proteomic analyses and 40 to the transcriptomic analyses. For the proteomic experiments, only the conditioned culture medium was collected from the respective cell cultures; the cells themselves were subsequently utilized for additional experimental purposes, thereby ensuring maximal use of biological material.

Cells were isolated from the hippocampi of newborn (0–2‐day‐old) BALB/c mice (RRID:MGI:2161072) of both sexes, following the methods previously described (Hajka et al. 2024). The protocol adhered to the standards of the EU Directive 2010/63/EU on animal experiments and received approval from the II Local Scientific Research Ethics Committee at the Wrocław University of Environmental and Life Sciences (approval no WNB.464.2.2020.IR). For the primary cell culture, hippocampi dissected from animals of a single litter were pooled together before plating. Typically, 6–8 newborns were used for one primary culture. A biological replicate refers to independently obtained cell cultures from different litters of mice.

After isolation, astrocytes were cultured in astrocyte culture medium (DMEM with 1 g/L glucose, 3.7 g/L sodium bicarbonate, 0.11 g/L sodium pyruvate and 94 mg/L D‐valine, supplemented with 10% Fetal Bovine Serum (FBS), 100 U/L penicillin, 0.1 mg/L streptomycin, 2 mM glutamine) and passaged every 8–10 days for a maximum of 4 times. For all experiments, astrocytes were seeded at a density of 50 000 cells/cm^2^. Two days before each experiment, the astrocyte culture medium was replaced with neuronal culture medium consisting of Neurobasal A (ThermoFisher Scientific, Waltham, MA, USA, A2477501), 2% B27 Supplement (ThermoFisher Scientific, Waltham, MA, USA, 17504044), 0.5 mM glutamine (Merck KGaA, Darmstadt, Germany, G7513), 12.5 μM glutamate (Merck KGaA, Darmstadt, Germany, G1251), 1% penicillin/streptomycin (bio‐west, Bourg, France, L0022‐100), to minimize the influence of different media types and to eliminate extracellular vesicles present in FBS, which is a component of the astrocyte medium. In the Neurobasal A‐based medium, B27 is used instead of FBS and it does not contain any vesicles. This is also the standard medium used for neuronal monoculture and astrocyte‐neuron co‐cultures.

Hippocampal neurons were isolated and cultured as described in (Mozrzymas et al. 2011), with a minor modification: the glucose concentration in the culture medium was adjusted to 2.5 mM. The procedure is described in detail in (Hajka et al. 2024). Cells were seeded at a density of 50 000 cells/cm^2^ and cultured for 14 days for full development of neuronal connections/protrusions. Pure neuronal monocultures were obtained by adding 2.5 μM Ara‐C to the medium 48 h after cells' isolation. The purity of the monocultures was verified by immunodetection of Map2 (neuronal marker) and Gfap (glial marker).

For EVs proteomic analysis, astrocyte‐neuron co‐culture was prepared. Astrocytes (10 000 cells/cm^2^) were introduced into a 14‐day‐old neuronal culture and the cells were cultured together for another 48 h.

### 2.2 Isolation of Neural Extracellular Vesicles

Extracellular vesicles were isolated from the conditioned media collected from cell cultures as described in (Gizak et al. 2020). Astrocytic extracellular vesicles (AEVs) were isolated 48 h after replacement of astrocytic media with neuronal media (as described above). In the case of neuronal extracellular vesicles (NEVs) isolation, conditioned medium was taken from 14‐day‐old cell cultures (the medium was not changed during the entire culture period). For the isolation of EVs from astrocyte‐neuron co‐culture (COEVs) used in LC–MS/MS experiment, the culture medium was harvested 48 h after introduction of astrocytes into the neuronal culture. The collected media were centrifuged to remove dead cells and membrane debris: first at 3000 × g for 10 min at 4°C, followed by 10 000 × g for 20 min at 4°C, with the pellet being discarded in both cases. The supernatant was then centrifuged at 100 000 × g for 70 min at 4°C. After the ultracentrifugation, the supernatant was removed, and the pellet (containing the extracellular vesicles) was resuspended in sterile PBS and washed two times (repeating the ultracentrifugation step after each wash). After the last wash, EVs were resuspended in a fresh portion of neuronal culture medium and applied to cultures of opposite cell types for 2 h (for the NGS experiment) or resuspended in lysis buffer (0.1 M Tris; 2% SDS; 50 mM DTT) for LC–MS/MS. In the NGS experiment, the suspension of EVs was always added to cell cultures at a 1:1 ratio (i.e., EVs isolated from 10 mL of conditioned culture medium collected from one 75 cm^2^ culture flask were applied to cells cultured in one 75 cm^2^ culture flask). EVs isolated from 1 mL of conditioned culture medium contained approximately 42 μg of protein and 30 pg of miRNA. EVs were always used for experiments immediately after their isolation. The culture medium used in the experiments contained B27 instead of serum, ensuring the absence serum‐derived vesicles.

### 2.3 Transmission Electron Microscopy

The samples were prepared using the negative staining technique. 5 μL drop of the solution was applied on glow‐discharge formvar–carbon film coated 400 mesh copper grids. After 5 min of incubation, an excess of the material was blotted. 1% uranyl acetate was applied for 1 min. The samples were allowed to dry. Imaging was performed using a transmission electron microscope (TEM) Jeol JEM F‐200 at a voltage of 200 kV. The modified method was previously described in (Choromańska et al. 2024).

### 2.4 Proteomic Analysis

Extracellular vesicles were isolated from conditioned media collected from astrocyte monocultures, neuron monocultures, and astrocyte‐neuron co‐cultures. The vesicles were purified by two rounds of PBS washing and centrifugation, and were lysed in the lysis buffer (as described above in Section 2). WF‐assay was applied in order to determine total protein and peptide concentration (Wiśniewski and Gaugaz 2015). The lysates were processed by the MED‐FASP method (Wiśniewski 2019) with a two‐step consecutive protein digestion using Lys‐C and trypsin (Wiśniewski et al. 2020). The generated peptides were analyzed by LC–MS/MS as described previously (Harwood et al. 2023). Analysis of peptide mixtures was performed using a QExactive HF‐X mass spectrometer (Thermo‐Fisher Scientific, Palo Alto). Aliquots containing 1 μg of total peptide were chromatographed on a 50 cm column with 75 μm inner diameter packed C18 material. Peptide separation was carried out at 300 nL/min for 95 min using an acetonitrile gradient of 5%–30%. The temperature of the column oven was 55°C. The mass spectrometer operated in data‐dependent mode with survey scans acquired at a resolution of 60 000. Up to the top 15 most abundant isotope patterns with charge ≥ +2 from the survey scan (300–1650 m/z) were selected with an isolation window of 1.4 m/z and fragmented by high‐energy collisional dissociation with normalized collision energies of 25 eV. The maximum ion injection times for the survey scan and the MS/MS scans were 20 and 28 ms, respectively. The ion target value for MS1 and MS2 scan modes was set to 3 × 106 and 105, respectively. The dynamic exclusion was 30 s. The spectra were analyzed using MaxQuant software (https://maxquant.net/maxquant/). Protein concentrations were calculated by “Total Protein Approach” (TPA) (Wiśniewski et al. 2012; Wiśniewski and Rakus 2014).

For further analysis, the R programming language was used. Functional analysis was performed with pathway enrichment method. Proteins discussed in the context of specific functional categories in Sections 3–7 were assigned based on enrichment analysis, which was further validated through a comprehensive literature review.

Due to the impossibility of separating individual biological replicates, as the research material was collected successively from multiple independent cell cultures obtained from different litters of animals, the measurements were performed as a single replicate. Cell cultures obtained from a single biological replicate (animals from one litter) provided an insufficient amount of EV proteins for LC–MS analysis. The analysis used EVs collected from cell cultures derived from a total of 100 individuals for each culture condition.

### 2.5 RNA Isolation

Total RNA was isolated from monocultures of astrocytes and neurons seeded into 75 cm^2^ culture flasks (50 000 cells/cm^2^) using the RNeasy Mini Kit (Qiagen, 74104) according to the manufacturer's protocol. The DNA digestion step was performed using the RNAse‐Free DNase Set (Qiagen, 79254). RNA was eluted in 30 μL of RNase‐free water. The isolated RNA was stored at −80°C until further analysis. The concentration and purity of RNA were measured using a NanoDrop 1000 spectrophotometer (Thermo Scientific; RRID:SCR_016517), RNA concentration was determined based on absorbance values at 260 nm. RNA was considered pure if the A260/A280 ratio was ~2.0 and the A260/A230 ratio ranged between 1.8 and 2.2. The next step involved assessing RNA quality by determining the RNA Integrity Number (RIN) using capillary electrophoresis performed using the Agilent 2100 Bioanalyzer (RRID:SCR_018043) and Agilent RNA 6000 Kit (Agilent Technologies, 5067‐1511). RNA was considered of good quality if the RIN value was > 8.0.

### 2.6 Next‐Generation Sequencing

Sequencing was performed on RNA isolated from astrocyte and neuron monocultures under control conditions and in the presence of extracellular vesicles derived from the opposite cell type, with three biological replicates (independently obtained cell cultures from different litters of mice). Next‐Generation Sequencing was conducted following the method described in (Sheriff et al. 2022). DNA libraries were prepared using the KAPA HyperPrep Kit (Roche, 07962347001), and sequencing was carried out on the NextSeq 500/550 instrument (Illumina Co), using the High Output Kit v2.5 (150 cycles) (Illumina Co, 20024907) according to the manufacturer's protocol. The obtained sequencing data were used for bioinformatic analysis.

### 2.7 RNA‐Seq Data Analysis

The sequences were obtained in FASTQ format, with automatic demultiplexing. Data was assessed for read quality using the FastQC tool (RRID:SCR_014583) [https://www.bioinformatics.babraham.ac.uk/]. Trimming of reads was not necessary. Subsequently, reads were aligned to the mouse genome (GRCm39) using the HISAT2 tool (RRID:SCR_015530) (Kim et al. 2015). Obtained .bam files were converted to .sam files with SAMtools (RRID:SCR_002105) [http://www.htslib.org/] and quantified with FeatureCounts (RRID:SCR_012919) (Liao et al. 2014). Differential analysis was then performed using DESeq2 (RRID:SCR_015687) (Love et al. 2014) on pre‐filtered data (genes that had less than 10 reads in total were not included into the analysis). Outlier testing was not performed and no data points were excluded. Genes with significantly different expression between the control group and the experimental group (*p*adj < 0.05) were visualized using heatmaps. Multiple testing correction was performed using the Benjamini‐Hochberg (BH) method to control the False Discovery Rate (FDR). For graphical representation of the differences, DESeq2‐normalized data was transformed with *Z*‐score standardization according to the following equation:

$$z=\frac{x-\mu }{\sigma }$$

where: *x*—unstandardized variable (read count), *μ*—the mean of the statistical population, *σ*—the standard deviation of the statistical population.

Functional analysis was performed using Gene Set Enrichment Analysis (GSEA) (RRID:SCR_003199) (Subramanian et al. 2005). Genes were ranked considering their *p*‐values and fold changes according to the equation:

$$\text{RANK}=\text{SIGN}\left(\log2\mathrm{FC}\right)\;x-\left(\log10\left(p-\text{value}\right)\right)$$

Additionally, GO Enrichment Analysis from clusterProfiler (RRID:SCR_016884) was conducted, separately for upregulated and downregulated genes (Yu et al. 2012). Genes discussed within the context of specific functional categories presented in Sections 3, 3.1, 3.2, 3.3, 3.4, 3.5, 3.6, 3.7, 4, 4.1, 4.2, 4.3, 4.4, 4.5, 5, 6 were assigned based on their initial fit to specific GO BP terms and supported by findings from the literature. These genes are visualized on heatmaps to illustrate their expression profiles within the identified functional categories.

No statistical methods were used to predetermine the sample size in this study. However, the sample size applied in this study is comparable to that of prior publications (Hasel et al. 2017; Tyssowski et al. 2018).

All steps of the analysis were performed using Python or R programming languages.

## 3 Results

### 3.1 Characterization of EVs and Proteomic Profiling of Their Content

**FIGURE 1:**
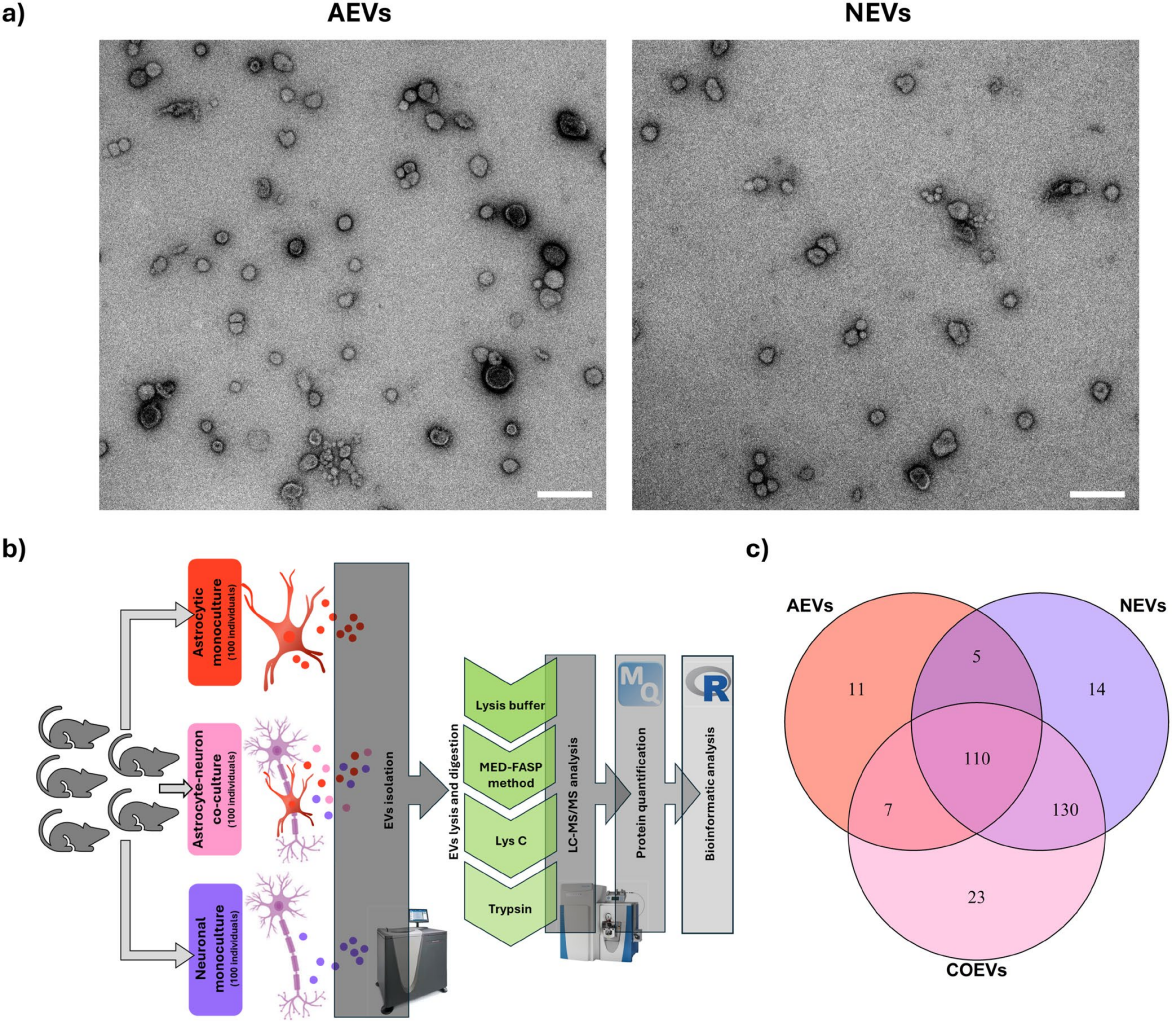
Characterization of extracellular vesicles secreted by astrocyte and neuron cultures. (a) Representative TEM images showing astrocyte‐ (left panel) and neuron‐derived (right panel) EVs. Scale bar = 200 nm. (b) LC–MS workflow: EVs were isolated from conditioned culture media of astrocytic and neuronal monocultures, as well as from astrocyte‐neuron co‐cultures. Following protein extraction, digestion, and purification, mass spectrometry was used to identify and quantify the EVs protein content. Bioinformatic tools were used for data analysis. (c) Venn diagram showing the overlap of proteins identified in EVs secreted by astrocytic monocultures (red), neuronal monocultures (purple), and astrocyte‐neuron co‐cultures (pink), *n* = 1 (pooled sample of conditioned medium collected from 20 independently prepared cell cultures, corresponding to a total of 100 animals per condition). AEVs, astrocytic extracellular vesicles; COEVs, astrocyte‐neuron co‐culture extracellular vesicles; NEVs, neuronal extracellulae vesicles.

We first characterized astrocyte‐ and neuron‐derived extracellular vesicles using transmission electron microscopy (TEM). Following our isolation protocol, we observed vesicle‐like structures with a well‐defined lipid bilayer. No amorphous or irregular structures indicative of protein aggregates or other contaminants were detected, confirming the purity of the preparations. The isolated EVs ranged from 15 to 280 nm in diameter, and their morphology was consistent with that commonly described for extracellular vesicles (Figure 1a). Further details regarding EVs characteristics are available in Figure S1.

Next, LC–MS/MS was used to analyze proteins present in extracellular vesicles isolated from astrocyte monocultures (AEVs), neuron monocultures (NEVs), and astrocyte‐neuron co‐cultures (COEVs). The study of EVs from co‐cultures was aimed at better understanding the physiological consequences of the physical interaction between these two cell types. The experimental design is shown in Figure 1b.

A limitation of this study is that the co‐culture medium contained vesicles secreted by both cell types and it was not possible to determine which proteins and at what concentrations originated from EVs of astrocytes versus neurons. However, the proteins uniquely detected in this condition provided valuable clues about the general changes in the physiology of the co‐cultured cells. Another limitation is that the amount of protein isolated from EVs from a single biological replicate was too small to analyze proteins by LC–MS/MS (1 mL of conditioned medium contained about 42 μg of protein). Therefore, biological replicates had to be pooled and analyzed together. However, since approximately 100 animals were used for each culture type, the proteomic analysis took into account individual variability. Due to the analysis of a single replicate, no differential analysis was conducted; only functional analysis of proteins contained in EVs.

In total, 679 proteins were identified, of which 300 were represented by more than one unique peptide detected and were subjected to further analysis. In astrocytic EVs, a total of 133 proteins were identified, 11 of which were unique to these culture conditions. In neuronal EVs, 259 proteins were identified, 14 of which were unique. In co‐culture EVs, there were 270 different proteins, with 23 being unique (Figure 1c).

Among proteins that were unique only for AEVs (meaning that physical interaction with neurons in co‐culture inhibited secretion of these proteins by astrocytes) were proteins involved in cell adhesion and mobility, regulation of axon elongation during central nervous system development, synaptic plasticity, and neuron regeneration (Cd9—CD9 antigen, Tnc—Tenascin, Itga7—Integrin alpha‐7), as well as proteins involved in transcription regulation (Hmgb1—High mobility group protein B1). Among proteins unique for NEVs (i.e., whose secretion into NEV was inhibited by co‐culture with astrocytes) were proteins involved in glucose and glycogen metabolism (Gsk3β—Glycogen synthase kinase 3 beta), lipid metabolism (Acsbg1—Long‐chain‐fatty‐acid‐CoA ligase ACSBG1), transcription regulation, and DNA repair (Ruvbl2—RuvB‐like 2, Fus—RNA‐binding protein FUS), translation regulation (Eprs1—Bifunctional glutamate/proline‐tRNA ligase), as well as the secretion of a GABA‐B receptor subunit protein (Kctd12—BTB/POZ domain‐containing protein KCTD12) and a protein regulating the stability of the GABA‐A receptor (Rac1—Ras‐related C3 botulinum toxin substrate 1).

Conversely, physical interactions of astrocytes and neurons in co‐cultures led to packaging of several proteins into COEVs that were not observed in EVs from monocultures. Among them were proteins involved in glucose and glycogen metabolism (Ugp2—UTP‐glucose‐1‐phosphate uridylyltransferase, Pygb—Glycogen phosphorylase), neurotransmitter production (Asrgl1—Isoaspartyl peptidase/L‐asparaginase), and proteins responsible for neuronal calcium dynamics (Atp2b2—Plasma membrane calcium‐transporting ATPase 2). However, the largest number of unique proteins in EVs isolated from co‐cultures was associated with proper brain development: axon growth, neuron differentiation, synapse morphology, and cell survival (Txnrd1—Thioredoxin reductase 1, Atp6ap2—Renin receptor, Tmem132a—Transmembrane protein 132A, Dpysl5—Dihydropyrimidinase‐related protein 5, Ncam1—Neural cell adhesion molecule 1, Eno2—Gamma‐enolase).

In addition, 68 of all identified proteins in our study were also listed in Vesiclepedia [http://microvesicles.org/, 04.10.2025] as the top 100 proteins found in EVs. Furthermore, in accordance with the Minimal Information for Studies of Extracellular Vesicles (MISEV; Welsh et al. 2024) we cross‐referenced our proteomic data against proteins considered as common markers of co‐isolated non‐EV structures. The comparative analysis between our dataset (proteins detected in EVs) and the MISEV reference lists is available in Table S1. Although some proteins flagged by MISEV as potential non‐EV contaminants were detected in our isolates, we interpret their presence as a reflection of their physiological role rather than insufficient purification. Numerous publications have reported the presence of such proteins, including ApoE, Igsf8, LDH, and HSP90, as established EV components with relevant biological roles in neural and different tissue types (Dean et al. 2005; Fan et al. 2023; Graner et al. 2009; Jiang et al. 2024; Kumar et al. 2024; Nikitidou et al. 2017; Tang et al. 2019; Yang et al. 2018; Zhang et al. 2025).

The complete set of proteins (including unique ones) observed in all three conditions is available in Supporting Information S1 (DOI: https://doi.org/10.34616/LERXTA).

Next, the functional analysis of EVs content was performed. Here, pathway enrichment analysis was used due to the impossibility of performing differential analysis. We included all proteins present in EVs from each culture type. As a background setlist we used all genes identified by next‐generation sequencing for the respective culture type from which the EVs were derived (RNA‐Seq results are presented in Section 3). This approach allowed for a comprehensive comparison of the protein functions represented in the EVs relative to the overall gene expression profile of the cells.

**FIGURE 2:**
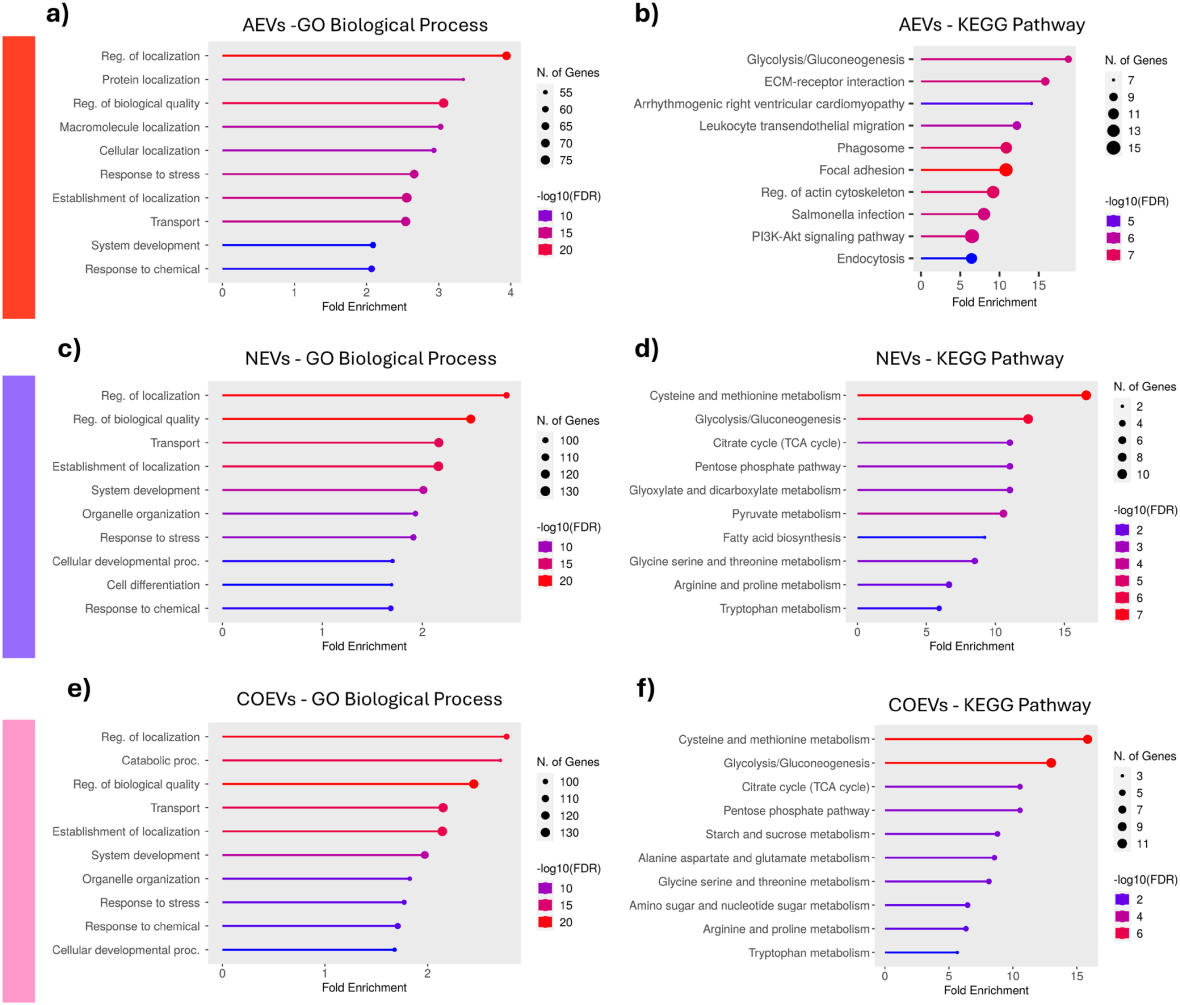
Functional characterization of the EV proteome. Analysis of enriched terms concerning Biological Process and KEGG Pathways for proteins contained in EVs originating from astrocytic (a, b) and neuronal (c, d) monocultures, as well as from astrocyte‐neuron co‐cultures (e, f); *n* = 1 (pooled sample of conditioned medium collected from 20 independently prepared cell cultures, corresponding to a total of 100 animals per condition). The circle size represents the number of genes, while the color represents false discovery rate (−log10(FDR)). Other GO terms (Cellular Component and Molecular Function) can be found in Supporting Information S2 (DOI: https://doi.org/10.34616/LERXTA). AEVs, astrocytic extracellular vesicles; NEVs, neuronal extracellular vesicles; COEVs, extracellular vesicles from astrocyte‐neuron co‐culture.

The extracellular vesicles derived from astrocytic monocultures were found to be enriched in proteins involved in biological processes such as the cellular response to stress and system development. Several categories also indicated a potential role of AEVs in regulation of protein localization (Figure 2a). Furthermore, KEGG pathway analysis suggested that AEV‐derived proteins are implicated in glycolysis and gluconeogenesis, regulation of the actin cytoskeleton, and PI3K‐Akt signaling, which plays a key role in the regulation of the cell cycle (Figure 2b).

NEVs were similarly enriched in proteins assigned to categories such as response to stress and system development, with additional terms related to cellular developmental processes and cell differentiation (Figure 2c). KEGG pathway analysis revealed the enrichment of several key metabolic pathways crucial for neuronal energy metabolism, including glycolysis and gluconeogenesis, the TCA cycle, the pentose phosphate pathway, pyruvate metabolism, and fatty acid biosynthesis (Figure 2d).

The terms identified for COEVs were consistent with those observed in both AEVs and NEVs, indicating a similar enrichment in proteins involved in development, stress response, and metabolic processes (Figure 2e,f).

### 3.2 Changes in the Concentrations of “Constitutive” EVs Proteins Induced by Co‐Culture of Neurons and Astrocytes

In this study, we demonstrated that EVs can modulate gene expression in both neurons and astrocytes, raising questions about the dynamic nature of astrocyte‐neuron communication. Specifically, it remained unclear whether the physical contacts between these two cell types and the ability to receive certain secretory signals influence the secretory profile of these cells. To explore this possibility, we investigated the proteomic profile of EVs secreted by astrocytes and neurons in co‐culture.

In Section 1, we showed that co‐culture of astrocytes and neurons modified the protein profile of extracellular vesicles, and we characterized the proteins constituting the unique cargo of COEVs. Here, we sought to determine whether co‐culture affected concentrations of proteins that were not unique but were found in both AEVs and NEVs (“constitutive” proteins). We focused on proteins related to the processes investigated in this paper, such as cellular development, synaptic transmission, stress response, and energy metabolism (keeping in mind the limitations of this analysis, described in Section 1).

**FIGURE 3:**
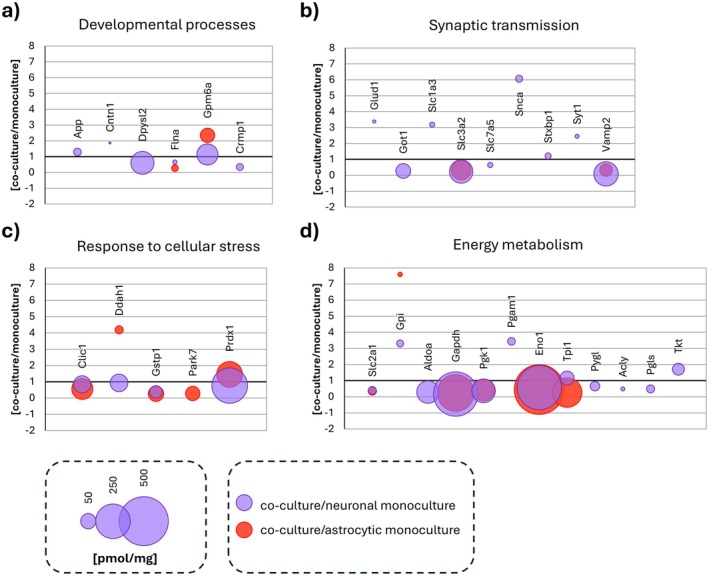
Changes in concentrations of proteins in EVs secreted by astrocytes and neurons in co‐culture. The changes are expressed as the difference between the concentration [pmol/mg] of proteins detected in EVs from the co‐culture and their concentration in EVs from astrocytic monoculture (red bubbles) or neuronal monoculture (purple bubbles). The size of the bubbles is proportional to the concentration of the identified proteins. Proteins are categorized based on the processes they are involved in: (a) developmental processes, (b) synaptic transmission, (c) response to cellular stress, and (d) energy metabolism; *n* = 1 (pooled sample of conditioned medium collected from 20 independently prepared cell cultures, corresponding to a total of 100 animals per condition).

In COEVs, increased concentrations of proteins involved in neurite outgrowth (App), axo‐glial junction formation (Cntn1), and neuronal differentiation (Gpm6a), and reduced concentrations of proteins associated with cell migration (Dpysl2, Flna, Crmp1) were observed (Figure 3a) compared to EVs from monoculture(s). Among these, Flna and Gpm6a were present in EVs from both neuronal and astrocyte monocultures, while the remaining proteins were exclusively secreted by NEVs.

In response to co‐culture, cells showed a reduced secretion into EVs of proteins associated with the production and uptake of excitatory neurotransmitters (Got1, Slc3a2, Slc7a5) and a key protein responsible for vesicle exocytosis (Vamp2). Conversely, there was an increase in secretion of proteins involved in the rapid clearance of neurotransmitters from the synaptic cleft (Glud1, Slc1a3), as well as proteins that modulate dopaminergic transmission (Snca) and regulate membrane interactions during synaptic vesicle trafficking at the active zone (Syt1) (Figure 3b).

Compared to both neuronal and astrocyte monocultures, co‐culture conditions resulted in a decreased secretion into EVs of proteins involved in the cellular stress response, including Clic1, Gstp1, and Park7. Proteins Ddah1 and Prdx1, which regulate the production of nitric oxide and hydrogen peroxide, respectively, exhibited distinct trends depending on whether their levels in COEVs were compared to those in NEVs or AEVs (Figure 3c). This variability arose from the fact that their concentrations in the co‐culture EVs approximated the median of the values observed in the monoculture EVs.

In the co‐culture EVs, a decrease in the levels of several proteins involved in energy metabolism (glycolysis, pentose phosphate pathway and glycogen metabolism) was observed, including Slc2a1, Aldoa, Gapdh, Pgk1, Eno1, Tpi1, Pygl, Acly, and Pgls. Conversely, an increase was noted for Gpi, Pgam1 and Tkt (Figure 3d).

### 3.3 Transcriptomic Profiling of EVs‐Treated Cells

Prior to gene expression analysis, we confirmed the uptake of EVs derived from the opposite cell type by astrocytes and neurons after 1 h of incubation. These observations, which validate effective EVs internalization, are presented in the Figure S2. We also determined that, from 1 mL of conditioned culture media collected from either astrocyte or neuronal monocultures, we can obtain EVs containing approximately 42 μg of protein and 30 pg of miRNA. For each experiment, the same amount of EVs was applied to the recipient cultures, using a 1:1 ratio: EVs isolated from 1 mL of conditioned medium were added to 1 mL of medium introduced to cell cultures. Cells were also always seeded in the same density as described in the Section 2 to maintain consistency.

**FIGURE 4:**
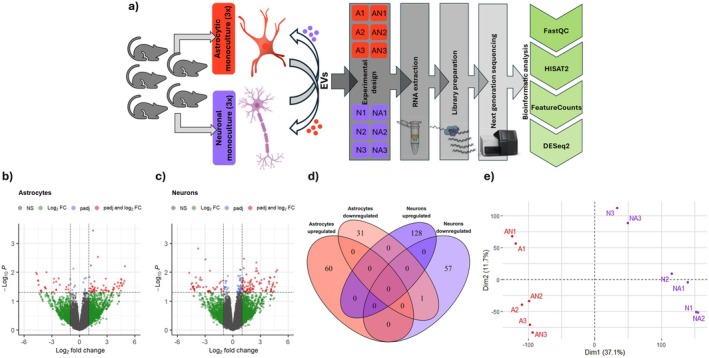
Transcriptomic analysis of untreated neurons and astrocytes and those cells incubated with extracellular vesicles (EVs). (a) Schematic representation of the experimental design: three independent biological replicates of neuronal (N) and astrocytic (A) monocultures were used as controls, and the experimental conditions included the incubation of these cells with EVs (NA and AN, respectively). RNA was extracted from both conditions, followed by sequencing and bioinformatics analysis using tools such as FastQC, HISAT2, FeatureCounts, and DESeq2. Volcano plots show differentially expressed genes (DEGs) between control (untreated cells) and experimental (EV‐treated cells) conditions for astrocytes (b) and neurons (c), respectively; *n* = 3 (number of independent cell culture preparations for both astrocytes and neurons). Red dots represent significantly regulated genes (adjusted *p*‐value < 0.05, log2FC > 1). (d) Venn diagram illustrating the overlap of differentially expressed genes between neuronal and astrocytic populations across different conditions. (e) Principal component analysis (PCA) plot displaying the clustering of individual samples based on the first two principal components. The distance between points reflects transcriptomic variability between samples. Numbers indicate the scores returned by the PCA analysis. NS, non‐significant; A, astrocytic monocultures; N, neuronal monocultures; AN, astrocytic monocultures incubated with neuronal extracellular vesicles; NA, neuronal monocultures incubated with astrocytic extracellular vesicles.

After confirming EV internalization and establishing standardized experimental conditions, we employed RNA sequencing to investigate the transcriptomic alterations in mouse hippocampal astrocytes and neurons induced by 2‐h incubation with extracellular vesicles isolated from the opposing cell type. We chose a 2‐h incubation period based on evidence showing that this timeframe is sufficient for EVs to be internalized by target cells and elicit measurable changes in gene expression (Bonsergent et al. 2021; Escrevente et al. 2011). The experiment included three biological replicates, each generated from independent cell cultures obtained from different mouse litters. This experimental approach allowed us to capture biological variability and explore the impact of cell‐type‐specific EVs on gene regulation. The detailed description of the experiment and data analysis is presented in Figure 4a.

In astrocytes, a total of 56 980 genes were identified based on the RNA‐Seq data, and 20 540 genes were taken for further analysis (genes with a total read count of < 10 were excluded). Our analysis revealed 92 genes with significant differences in expression (DEGs—differentially expressed genes) between the control group (untreated monoculture of astrocytes—A) and the experimental group (monoculture of astrocytes exposed to neuronal EVs—AN). Specifically, 60 genes were upregulated, and 32 genes were downregulated in response to the NEVs treatment (Figure 4b).

In neurons, we identified 56 980 genes in total, from which 24 868 were taken for further analysis. 186 genes showed differential expression between the control condition (untreated monoculture of neurons—N) and the experimental condition (monoculture of neurons exposed to astrocytic EVs—NA). Of these, 128 genes were found to be upregulated, whereas 58 genes were downregulated (Figure 4c). DEGs and all statistical parameters are presented in Supporting Information S3 (DOI: https://doi.org/10.34616/LERXTA). A full set of DEGs for astrocytes and neurons is also presented on heatmaps in Figure S3.

Figure 4d shows a Venn diagram illustrating the differential gene expression analysis results between two experimental conditions for astrocytes and neurons. There is almost no intersection between the two sets, meaning that there are few genes that exhibit significant differential expression in both astrocytes and neurons under the experimental conditions. In fact, we found only one gene downregulated in both cell types by EVs—the *Nemf* gene, encoding ribosome quality control complex subunit NEMF. This result indicates that the incubation with EVs led to cell type‐specific responses.

In the PCA plot shown in Figure 4e, the samples are clearly separated into two distinct clusters. The astrocyte samples are grouped together, demonstrating a similar gene expression pattern across the biological replicates. Conversely, the neuron samples form a separate cluster, indicating a different gene expression profile compared to astrocytes. This clear separation suggests that the RNA‐Seq data captured significant differences between the transcriptomes of astrocytes and neurons.

Additionally, clustering analysis was performed using both agglomerative hierarchical clustering and K‐means clustering methods. Supporting Information contain additional plots displaying the distance matrix and dendrograms (Figure S4). This analysis revealed that samples consistently cluster within their respective biological replicates, with cells derived from a single isolation being more similar to each other than to cells from other isolations, regardless of experimental conditions.

Functional analysis of gene set was carried out using both Gene Set Enrichment Analysis (GSEA) and GO Enrichment Analysis from clusterProfiler package and it showed no significant enrichment of any terms/pathways (*p*adj > 0.05). Results of these analyses are presented in Supporting Information S4 (DOI: https://doi.org/10.34616/LERXTA).

Since the functional analysis of RNA‐Seq did not identify any significant GO terms and since the terms related to developmental processes, response to stress, and energy metabolism were recurrent across the different EV groups, we examined changes in gene expression and the proteomic content of EVs in the context of these terms, aiming to better understand the molecular mechanisms underlying the observed enrichment in these processes. More information about enriched pathways and processes can also be found in Supporting Information S2 (DOI: https://doi.org/10.34616/LERXTA).

### 3.4 Differential Expression of Genes Involved in Developmental Processes Induced by EV Treatment

In the present study, most genes with altered expression between control (untreated) cells and cells exposed to EVs were related to developmental processes. This suggests a significant role of EV cargo in modulating cellular growth and development in both astrocytes and neurons. Therefore, we decided to take a closer look at DEGs and investigate whether there are any reports on their roles in the context of all investigated terms. A summary of genes, along with their functions and corresponding literature references, is provided in the Table S2.

**FIGURE 5:**
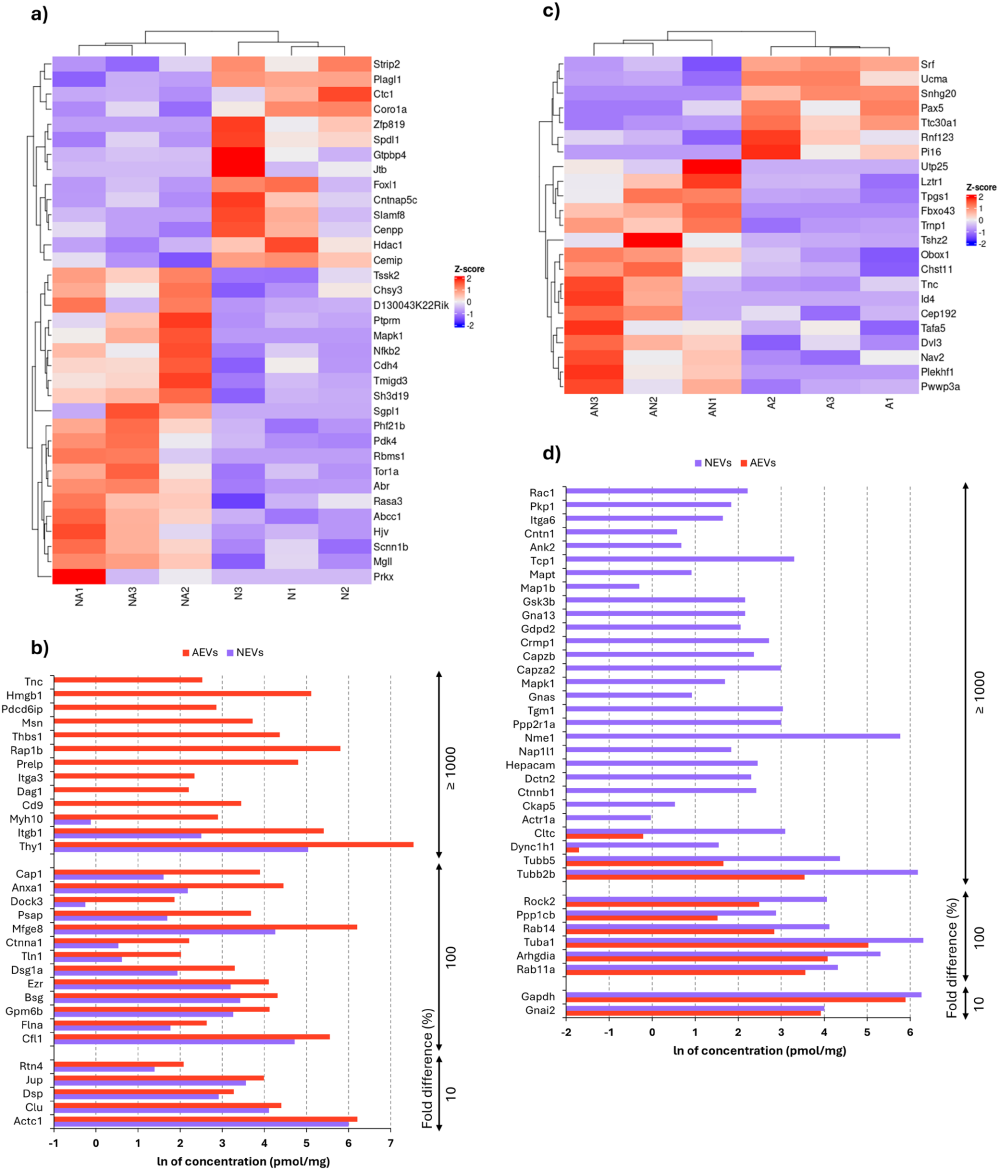
Differential gene expression in EV‐treated cells and protein concentration profiles of EVs. (a, c) Heatmaps showing differential gene expression profiles in neurons treated with AEVs (a) and astrocytes treated with NEVs (c); *n* = 3 (number of independent cell culture preparations for both astrocytes and neurons). Red indicates upregulation, and blue indicates downregulation of genes engaged in developmental processes. (b, d) Comparison of protein concentrations (expressed as natural logarithm, ln, of pmol/mg) identified in EVs. Fold differences are provided as percentages (%). Panel (b) shows the concentrations of proteins found in AEVs that may affect neuronal development compared to their concentrations in NEVs. These proteins are involved in adhesion, connection, polarity, motility, neurite growth, and differentiation. Only proteins with higher concentrations in AEVs compared to NEVs are shown. Panel (d) highlights concentrations of proteins found in NEVs that may impact astrocyte development, compared to their concentrations in AEVs. These proteins are involved in cell proliferation, cytoskeletal reorganization, cell growth, and adhesion. Only proteins with higher concentrations in NEVs compared to AEVs are displayed; *n* = 1 (pooled sample of conditioned medium collected from 20 independently prepared cell cultures, corresponding to a total of 100 animals per condition). A, astrocytic monoculture; N, neuronal monoculture; AN, astrocytes incubated with neuronal extracellular vesicles; NA, neurons incubated with astrocytic extracellular vesicles; AEVs, astrocytic extracellular vesicles; NEVs, neuronal extracellular vesicles.

In neurons exposed to AEVs, the expression of genes involved in progenitor migration (e.g., *Rbms1*, *Tor1a*), synapse formation (e.g., *Ptprm*), and differentiation (e.g., *Phf21b*, *Rbms1*) were increased, while the expression of a gene responsible for maintaining cellular proliferation (*Plag1*) was decreased (Figure 5a). These findings may suggest that astrocytic signals switch neuronal gene expression from progenitor‐like to mature neuron‐like. Proteomic analysis of AEV content supports this hypothesis since they contained numerous proteins engaged in neurogenesis (Figure 5b), such as proteins involved in cell adhesion (e.g., Ctnna1, Itga3), cell connection (e.g., Ezr, Msn), polarity (e.g., Anxa1), motility (e.g., Cfl1, Flna) and most importantly, proteins responsible for neurite growth (e.g., Gpm6b, Psap, Tnc) and differentiation (e.g., Clu). Since developmental processes provide the foundation for synaptic transmission and plasticity by establishing neural connectivity and functionality, some of the neuronal genes discussed in this section are also involved in the regulation of synaptic transmission, and their role in this process will be discussed in the section devoted to this topic.

On the other hand, in astrocytes exposed to NEVs, the expression of genes involved in spindle formation (e.g., *Cep192*), proliferation (e.g., *Trnp1*, *Id4*), and migration (e.g., *Tnc*) increased, while the expression of a negative regulator of cell division (*Srf*) decreased (Figure 5c). These findings highlight the significance of NEV cargo in regulation of astrocyte proliferation, a conclusion further supported by our additional immunocytochemical and cytometric studies (Figure S5). We observed that astrocytes incubated with neuron‐derived EVs had elongated protrusions and frequently occurred in clusters, which suggested that cell divisions had recently been completed (Figure S5a). This observation was consistent with the measurement of Ki‐67 levels in astrocyte nuclei, indicating a 44% increase in this proliferation marker after treatment with NEVs (Figure S5b). Furthermore, cell cycle analysis revealed an elevated proportion of astrocytes in the G2/M phase (Figure S5c). Proteomic analysis of NEVs content also confirmed their participation in glial development (Figure 5d). Among the proteins contained in NEVs, we identified a significant group involved in cell proliferation (e.g., Ctnnb1, Dctn2, Nap1l1, Rab11a), cytoskeletal reorganization (e.g., Capza2, Map1b, Rock2), as well as cell growth (e.g., Mapk1) and adhesion (e.g., Ank2, Cntn1, Ctnnb1).

These results suggest that EV‐mediated communication between astrocytes and neurons may be essential for the proper development of both cell types.

### 3.5 Alterations in Synaptic Transmission‐Related Genes Induced by EV Treatment

Intercellular communication between astrocytes and neurons plays an important role in modulating synaptic transmission and neural plasticity. Astrocytes contribute metabolically by providing energy substrates and recycling neurotransmitters, but they also modulate synaptic function through the release of gliotransmitters (Ota et al. 2013; Pellerin and Magistretti 2012).

We explored the possibility that astrocyte‐neuron communication via the secretion of EVs might influence neuronal synaptic transmission. We found that the incubation of neurons with AEVs influenced the expression of genes involved in synapse maturation (*Hdac1*) as well as a range of proteins regulating proper LTP progression or short‐term plasticity (*Phf21b*, *Pfdn5*, *Nfkb2*, *Sgpl1*). In the AEVs proteome, we also found synaptic plasticity regulators, such as Rtn4 and Tnc.

In astrocytes treated with NEVs, changes in the expression of genes assigned to the category of “synaptic transmission” were also observed. However, a thorough analysis of the literature data revealed that proteins encoded by these genes are involved in typically neuronal processes, and in astrocytes, they play mainly developmental roles described in Section 3. The only gene of note outside this category was the gene encoding Gpr37, a protein expressed in most astrocytes that regulates NMDAR activation. All DEGs and their functions are presented in Table S2.

Our findings indicate that astrocyte‐derived factors transported in EVs can influence synaptic plasticity and neuronal transmission. However, it remains to be clarified whether these factors exert their effects by stimulating the maturation of synaptic transmission mechanisms in neurons or by directly modulating synaptic activity through regulation of inhibitory or stimulatory mechanisms.

### 3.6 Cellular Stress Response‐Related Gene Changes Induced by EV Treatment

Recently, we discovered that neuronal EVs can affect both total and mitochondrial calcium levels in astrocytes, leading to a slight decrease in mitochondrial membrane potential (Hajka et al. 2024). While this may have important implications for signal transduction and activation of specific pathways, it may also affect the cellular redox state, as reactive oxygen species (ROS) production is closely related to calcium levels (Görlach et al. 2015). Surprisingly, despite the changes in calcium concentrations, both total and mitochondrial ROS levels remained unchanged in the presence of NEVs (Hajka et al. 2024). This finding prompted us to investigate the potential influence of EVs on the expression of genes related to the cellular stress response.

**FIGURE 6:**
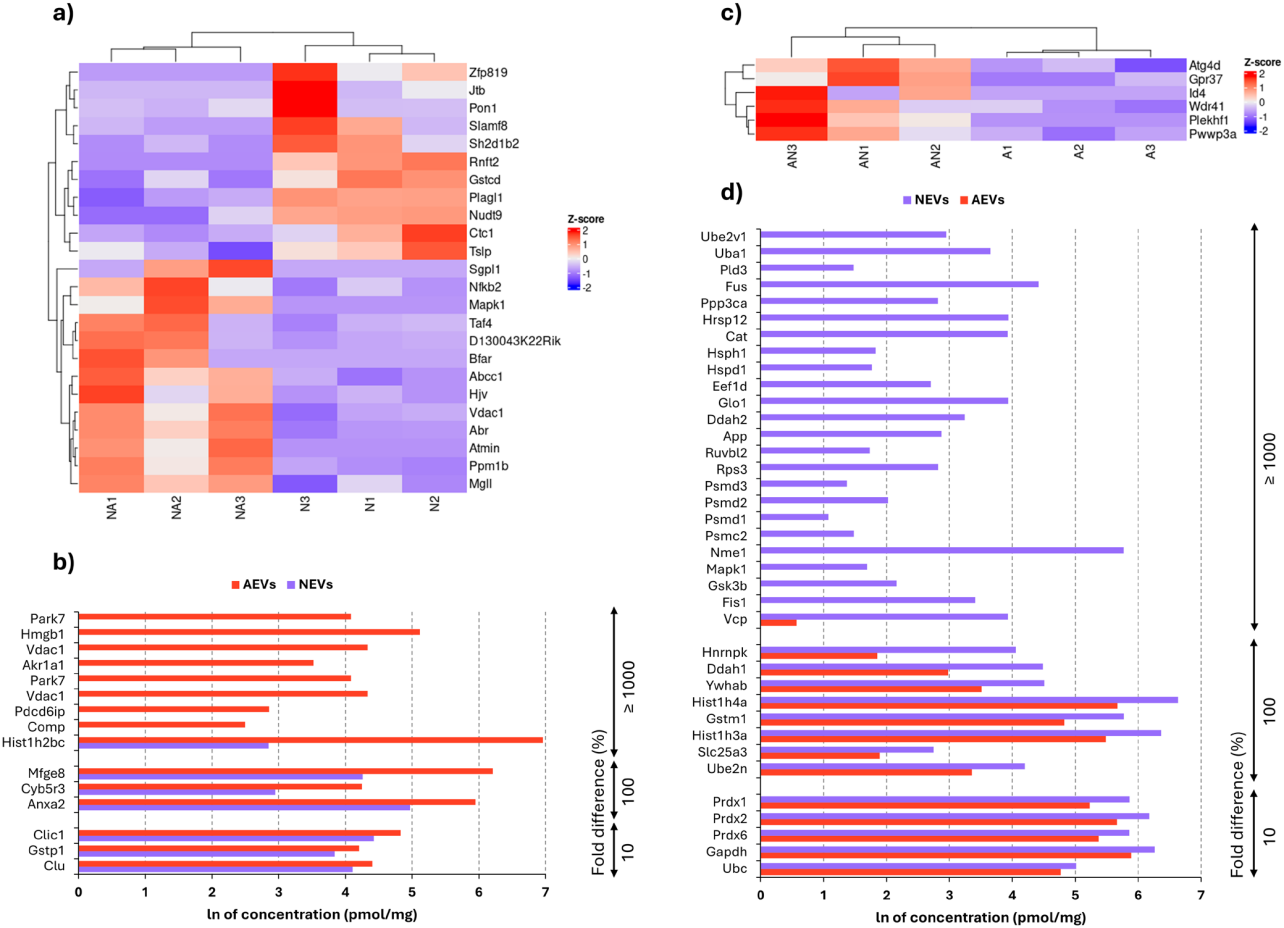
Differential gene expression in EV‐treated cells and proteins concentration profiles of EVs. (a, c) Heatmaps showing differential gene expression profiles in neurons treated with AEVs (a) and astrocytes treated with NEVs (c); *n* = 3 (number of independent cell culture preparations for both astrocytes and neurons). Red indicates upregulation, and blue indicates downregulation of genes engaged in developmental processes. (b, d) Comparison of protein concentrations (expressed as natural logarithm, ln, of pmol/mg) identified in EVs. Fold differences are provided as percentages (%). Proteins involved in apoptosis regulation, oxidative stress response, metabolism of toxic substances, mitochondrial response to cellular stress, DNA repair and heat‐shock proteins are shown. Panel (b) presents the concentrations of proteins identified in AEVs that may influence the neuronal stress response, compared to their concentration in NEVs. Only proteins with higher concentrations in AEVs compared to NEVs are shown. Panel (d) highlights concentrations of proteins found in NEVs that may impact astrocyte stress response, compared to their concentrations in AEVs. Only proteins with higher concentrations in NEVs compared to AEVs are displayed; *n* = 1 (pooled sample of conditioned medium collected from 20 independently prepared cell cultures, corresponding to a total of 100 animals per condition). A, astrocytic monoculture, N, neuronal monoculture; AN, astrocytes incubated with neuronal extracellular vesicles; NA, neurons incubated with astrocytic extracellular vesicles; AEVs, astrocytic extracellular vesicles; NEVs, neuronal extracellular vesicles.

In response to factors contained in AEVs, neurons altered expression of genes involved in protection against oxidative stress (*Pon1*, *Slamf8*, *Nudt9*, *Nfkb2*, *Atmin*, *Ppm1b*) and apoptosis (*Sgpl1*, *Mapk1*, *Bfar*, *Vdac1*), as well as those engaged in mitophagy (*Tslp*) and regulating the response to glutathione (*Abcc1*) (Figure 6a). Astrocytes responded to neuronal factors by adjusting expression of genes related to autophagy and apoptosis (*Atg4d*, *Id4*, *Wdr41*, *Plekhf1*), as well as DNA repair (*Pwwp3a*) (Figure 6c). In both astrocytic and neuronal EVs, we discovered proteins regulating apoptosis (in AEVs: Clu, Pdcd6ip; in NEVs: Ywhab, Ruvbl2), oxidative stress (in AEVs: Gstp1, Park7; in NEVs: Gstm1, Prdx1), heat‐shock proteins (in AEVs: Anxa2; in NEVs: Hspd1, Hsph1), proteins responsible for toxic substances metabolism (in AEVs: Akr1a1; in NEVs: Cat), mitochondrial response to cellular stress (in AEVs: Vdac1; in NEVs: Ppp3ca), and DNA repair (in AEVs: Hmgb1, Hist1h2bc; in NEVs: Fus, Hnrnpk) (Figure 6b,d).

### 3.7 Alterations in Genes Involved in Energy Metabolism Induced by EV Treatment

Given that maintaining energy homeostasis is a critical aspect for brain functioning, and it is known that this balance is achieved through communication between astrocytes and neurons (Pellerin and Magistretti 2012), we investigated whether there are changes in the expression of genes involved in energy metabolism in these cells under the influence of EVs.

**FIGURE 7:**
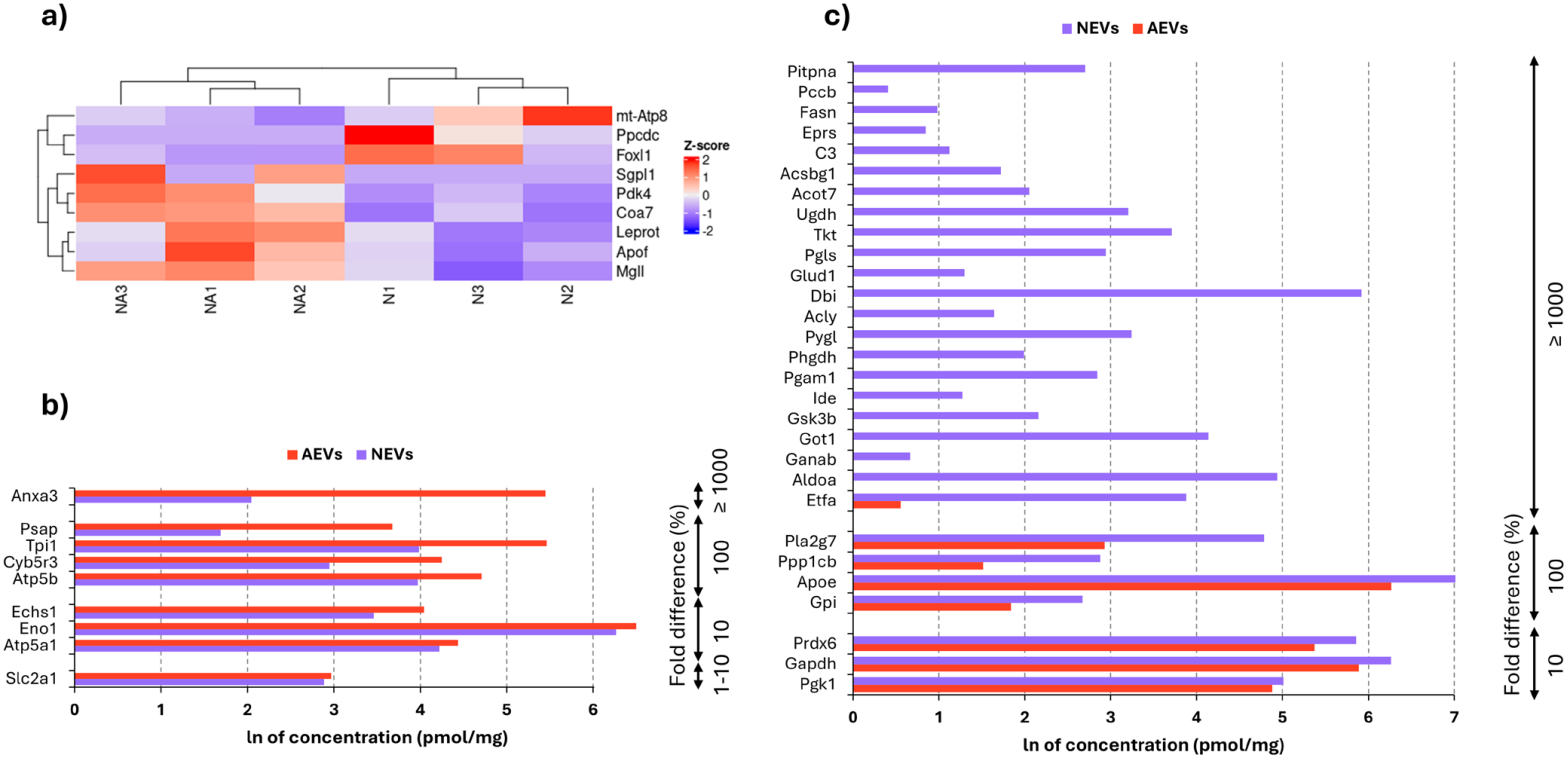
Differential gene expression in EVS and proteins concentration profiles of EVs. (a) Changes in gene expression involved in energy metabolism in neurons following incubation with AEVs; *n* = 3 (number of independent cell culture preparations for both astrocytes and neurons). Red indicates upregulation, and blue indicates downregulation of genes. (b, c) Comparison of protein concentrations in EVs, presented as the natural logarithm (ln) of pmol/mg, with fold differences expressed as percentages (%). The analysis includes proteins associated with energy metabolism. Panel (b) highlights proteins enriched in AEVs that may support neuronal energy metabolism, showing only those with higher concentrations in AEVs compared to NEVs. Panel (c) focuses on proteins enriched in NEVs that could affect the astrocyte energy metabolism, displaying only those with higher concentrations in NEVs than in AEVs; *n* = 1 (pooled sample of conditioned medium collected from 20 independently prepared cell cultures, corresponding to a total of 100 animals per condition). N, neuronal monoculture; NA, neurons incubated with astrocytic extracellular vesicles; AEVs, astrocytic extracellular vesicles; NEVs, neuronal extracellular vesicles.

In the present study, after incubation of neurons with AEVs we observed an altered expression of genes involved in glucose metabolism (*Pdk4*), oxidative phosphorylation (*Mt‐Atp8*, *Coa7*), coenzyme A synthesis (*Ppcdc*), and lipid homeostasis (*Sgpl1*, *Apof*, *Mgll*) (Figure 7a). Analysis of the protein content of AEVs revealed the presence of proteins involved in glucose metabolism (Eno1, Tpi1), oxidative phosphorylation (Atp5a1, Atp5b), and lipid metabolism (Psap, Echs1) (Figure 7b).

In astrocytes exposed to neuronal extracellular vesicles, a significant decrease in *Dlst* expression was observed, along with an increase in *Ndor1* expression. Dlst is a key enzyme in the tricarboxylic acid (TCA) cycle, where it catalyzes the transfer of succinyl groups to coenzyme A, leading to the production of succinyl‐CoA, which is crucial for ATP generation through oxidative phosphorylation (Brown et al. 2004). In contrast, Ndor1 plays a role in redox reactions, facilitating electron transfer and maintaining redox homeostasis (Finn et al. 2005). Although changes in the expression of genes involved in energy metabolism in astrocytes were not statistically significant, NEVs contained a number of energy metabolism proteins (Figure 7c). These proteins are involved in glucose (Aldoa, Gapdh, Gpi, Pgam1, Pgk1, Phgdh) and glycogen (Gsk3b, Pygl) metabolism, Krebs cycle (Acly, Dbi), pentose phosphate pathway (Pgls, Tkt), and lipid metabolism (Acsbg1, Apoe, Fasn).

Taken together, these observations may indicate that EV‐dependent intercellular communication actively shapes cellular metabolism and plays a role in sensing and responding to neuronal energy demands.

## 4 Discussion

Intercellular communication between astrocytes and neurons plays an important role in practically all functions of nervous system. Astrocytes play pivotal roles in neurogenesis and brain development. They regulate neural stem cell proliferation by secreting growth factors which promote the expansion and differentiation of neural progenitor cells (Martinez and Gomes 2002; Reuss et al. 1998). During neuronal migration, astrocytes provide crucial structural support and guidance, ensuring that neurons reach their correct destinations (Lois et al. 1996). They also contribute to synaptogenesis and synaptic remodeling by releasing signaling molecules and clearing excess neurotransmitters and synaptic debris (Baldwin and Eroglu 2017; Haroon et al. 2017). Additionally, astrocytes maintain the extracellular environment by regulating ion balance, pH, and neurotransmitter levels, thus supporting stable neuronal function (Theparambil et al. 2020). They modulate neuroinflammation, which can impact neurogenesis and brain development, and help maintain the integrity of the blood–brain barrier by interacting with endothelial cells (de Rus Jacquet et al. 2023). Furthermore, astrocytes contribute metabolically by providing energy substrates and recycling neurotransmitters, and influence neuronal activity through the release of gliotransmitters and modulation of synaptic transmission and plasticity, affecting learning, memory, and overall brain function (Ota et al. 2013; Pellerin and Magistretti 2012) However, less is known about the influence of neuronal signals on astrocyte development and function.

In this study, we explored the physiological dynamics of the astrocyte–neuron relationship, focusing specifically on extracellular vesicle‐mediated communication. We identified proteins secreted in EVs derived from astrocytic and neuronal monocultures and compared them with proteins present in EVs released by astrocyte–neuron co‐culture, which more closely approximate the physiological state. Furthermore, we examined transcriptomic changes in astrocytes and neurons following exposure to EVs from the opposite cell type to determine which cellular processes are modulated by this bidirectional communication.

### 4.1 EV‐Carried Proteins Orchestrate Developmental Programs of Neural Cells

Among the various components of EVs, miRNA is the most studied molecule for the regulation of gene expression because it modulates posttranscriptional mRNA levels by either degrading the mRNA or inhibiting its translation. Analyzing the overall miRNA content in EVs is challenging due to the limited sensitivity of current techniques in relation to the matrix amount. In contrast, proteomic profiling of EVs provides complementary and equally valuable insights, since many EVs proteins are established transcription factors, RNA binding proteins, or key signaling molecules.

For proteomic analysis, EVs were isolated from conditioned media of astrocytic monocultures (collected 48 h after the last medium change, with astrocytes maintained in neuronal medium), neuronal monocultures (collected 14 days after plating, without medium exchange), and astrocyte–neuron co‐cultures (collected 48 h after astrocyte addition to neuronal cultures). These collection time points reflect cell‐specific requirements: astrocytes, being proliferative, require regular nutrient replenishment, whereas neurons rely on matrix‐associated secreted proteins and are sensitive to medium replacement. The co‐culture setup, involving direct cell–cell contacts, allowed secretion profiles to stabilize while remaining consistent with the AEV collection timeframe.

Among the proteins that regulate gene expression, we identified Hnrnpk in all types of EVs—AEVs, NEVs, and COEVs. Hnrnpk is an RNA‐binding protein involved in axonogenesis. Suppressed expression of Hnrnpk in Xenopus embryos has been shown to result in aberrant microtubules, microfilaments, and neurofilaments. Hnrnpk has also been found to post‐transcriptionally regulate Arp2, tau, and α‐internexin. Notably, simultaneous knockdown of all three genes led to an axonless phenotype (Liu and Szaro 2011).

Hmgb1, a nuclear nonhistone protein involved in gene transcription regulation, was found exclusively in AEVs. Hmgb1 enhances neurite outgrowth in vitro and is associated with proliferation and differentiation of progenitor cells in vivo (Zhao et al. 2020).

Sfpq was observed in both NEVs and COEVs, as its expression is specific to neurons. It is an RNA‐binding protein that maintains transcriptional elongation of long genes. Disruption of Sfpq leads to neuronal apoptosis in the developing mouse brain due to downregulation of genes essential for proper development (Takeuchi et al. 2018).

In turn, Fus protein was found exclusively in NEVs. Fus is a well‐known RNA‐binding protein, whose mutations are often associated with neurodegenerative disorders such as amyotrophic lateral sclerosis (ALS) and frontotemporal dementia (FTD). Although Fus protein is normally localized in the cell nucleus, as the disease progresses it can move into the cytoplasm and form stress granules. Studies have shown that Fus interacts with Matr3, and mutations in Matr3 have been identified as another cause of ALS (Kamelgarn et al. 2016). Notably, we detected Matr3 in both NEVs and COEVs.

We also identified numerous proteins directly involved in cellular processes, as listed in the Results section. Taken together, this suggests that the content of both AEVs and NEVs supports developmental processes, which is consistent with the results of our NGS study discussed later in this section.

### 4.2 Intercellular Cross‐Talk Reshapes the Proteomic Landscape of EVs

Proteomic analysis of EVs from co‐cultures of astrocytes and neurons aimed to determine to which extent cells adjust the content of EVs in response to feedback signals. We observed both significant increases and decreases in specific protein levels in EVs from co‐cultures compared to EVs from monocultures, highlighting that interactions between these cells dynamically regulate EV cargo. Although this experiment did not differentiate between cargo of AEVs and NEVs, the data on proteins that were uniquely detected in this condition or disappeared from the COEVs provided valuable clues about the general changes in the physiology of the co‐cultured cells.

Exclusively in COEV, we identified several proteins linked to development as well as synaptic activity. Among them was Asrgl1, which is likely involved in excitatory neurotransmitter L‐aspartate formation and is associated with retinal photoreceptor degeneration in knockout mice and retinitis pigmentosa in humans (Zhou et al. 2022). Atp2b2, crucial for calcium homeostasis, is linked to neurodevelopmental disorders, ataxia, intellectual disability, autism, seizures, and epilepsy (Poggio et al. 2023). Ugp2 and Pygb, regulating glycogen synthesis and breakdown respectively, indicate active energy metabolism.

Taking the results from the COEV analysis together, physical interactions between astrocytes and neurons stimulate the secretion of proteins in EVs that may regulate synaptic transmission by modulating neurotransmitter synthesis and calcium homeostasis. Additionally, our observations suggest that these cellular interactions promote both glycogen synthesis and breakdown, indicating an increased energy demand in astrocytes and the need to store energy in the form of glycogen to better support neuronal metabolism. Overall, the results of the COEV proteomic analysis reinforce the view that EV cargo influences both synaptic function and cellular energetics.

### 4.3 EVs Induce Cell‐Type Specific Changes in Gene Expression

Knowing that physical contact between astrocytes and neurons alters the protein profile of EV cargo, we next asked whether EVs alone can influence the cellular transcriptome and, if so, which aspects of cell physiology are affected. Although we are aware that monocultures do not fully recapitulate the physiological state, they are nevertheless routinely used as in vitro models and provide a valuable system for studying interactions between these cell types.

We selected a 2‐h incubation period for astrocytes or neurons with EVs derived from the opposing cell type. This choice was based on a preliminary RNA‐seq analysis, which indicated that the most pronounced transcriptomic changes occurred 2 h after EV treatment. We confirmed that this duration was sufficient for EVs uptake (Figure S1) and for eliciting measurable changes in gene expression (Hajka et al. 2024). While the observed transcriptomic changes in recipient cells likely reflect the functional impact of EVs' protein and miRNA cargo, we cannot exclude the possibility that EV‐delivered mRNAs also contributed to the transcriptome alterations detected in this study.

The RNA‐Seq analysis showed that both NEVs and AEVs induce significant, cell‐type‐specific changes in gene expression. Notably, among the genes affected by EVs, *Nemf* was the only one commonly regulated in both astrocytes and neurons, highlighting the specificity of cellular responses to EV cargo. Collectively, astrocytes mainly respond to NEVs by increasing proliferation and migration, suggesting their potential role in supporting glial development in the nervous system, while neurons respond to AEVs by enhancing differentiation and synapse formation, suggesting that astrocytes are crucial for the developmental transition of neuronal progenitors. The modulation of genes linked to synaptic transmission indicates that AEVs may influence long‐term potentiation and adaptive neuronal processes, highlighting their key role in neuron–glia communication.

### 4.4 Impact of NEVs on Astrocyte Expansion and Glycolytic Shift at the Transcriptomic Level

In astrocytes exposed to NEVs, *Cep192*, *Trnp1*, *Tnc*, *Id4*, *Dvl3*, and *Lztr1* genes were upregulated. Cep192 is a protein critical for mitotic spindle formation, with its depletion reducing motility and altering cell polarization, influencing thus glia development (O'Rourke et al. 2014). Trnp1 regulates cerebral cortex expansion, neural stem cell self‐renewal, influencing number of intermediate progenitors and basal radial glial cells (Stahl et al. 2013). Tnc controls astroglial progenitor division and migration (Karus et al. 2011), while Id4 promotes astrocyte proliferation in response to neuronal death (Lee et al. 2011). Dvl3 modulates Wnt signaling, and Lztr1 regulates the RAS/MAPK pathway, with its deficiency increasing Gfap expression (Kafka et al. 2019). These transcriptomic changes align with our immunocytochemical and flow cytometric results, confirming NEVs' role in promoting astrocyte proliferation and migration.

Furthermore, the transcriptomic analysis results suggest that under the influence of neuronal EVs, astrocytes undergo significant metabolic changes. We observed a decrease in *Dlst* gene expression, which may indicate reduced involvement of the TCA cycle in astrocytes and a potential shift toward glycolysis as the primary source of ATP generation in astrocytes (log2FC: *Hk1* = 0.14, *Pfkm* = 0.56, *Aldoa* = 1.2, *Gpd2* = 0.35, *Pgam1* = 0.31, *Pgk1* = 0.36, *Eno1* = 0.28, *Pkm* = −0.08, *Ldhc* = 1.34; *p*adj > 0.05 in all cases), consistent with the astrocyte‐neuron lactate shuttle (Mamczur et al. 2015; Pellerin and Magistretti 2012). The increase in *Ndor1* expression may promote the maintenance of redox homeostasis in astrocytes, which is important for sustaining glycolysis and lactate production. This is in line with our recent results showing that NEVs cargo reduces (at the protein level) the astrocytic level of a regulatory enzyme of gluconeogenesis, fructose 1,6‐bisphosphatase (Fbp2), but increases the level of some glycolytic enzymes (Hk2, Pfkm), glucose (Glut‐1), and lactate (Mct4) transporters, as well as lactate release. These changes were observed after 48 h incubation of astrocytes with NEVs and confirmed by immunocytochemistry (Hajka et al. 2024).

### 4.5 AEV‐Driven Transcriptomic Regulation of Neuronal Development and Synaptic Function

In neurons exposed to AEVs, we observed increased expression of genes involved in neural progenitors' division and their differentiation to neurons during neocortex development (*Rbms1*) (Habib et al. 2022), synapse formation (*Ptprm*) (Mo et al. 2022), proliferation‐differentiation switch of neurogenesis in the developing brain (*Phf21b*) (Basu et al. 2020), and required for a proper pattern of neuronal migration during development (*Tor1a*) (McCarthy et al. 2012), as well as for adult neurogenesis (*Mgll*) (Syal et al. 2020). AEVs also upregulated genes associated with synaptic plasticity, including *Phf21b*, *Pfdn5*, *Sgpl1*, and *Nfkb2*. Phf21b protein contributes to memory formation (Chin et al. 2022); Pfdn5 is involved in LTP and neurodegeneration (Kadoyama et al. 2019); Sgpl1 influences presynaptic structure and hippocampal plasticity (Mitroi et al. 2016); Nfkb2 is critical for memory consolidation via PKA‐CREB signaling (Kaltschmidt et al. 2006).

On the other hand, in neurons exposed to AEVs, we observed a decrease in *Plag1* and *Hdac1* gene expression. Plag1 is a transcription factor associated with neurodevelopment. Loss of Plag1 has been shown to reduce the proliferation of neocortical progenitors (Gasperoni et al. 2024). In turn, the activity of Hdac1 results in chromatin condensation and transcriptional repression of genes related to neurogenesis. Deletion of Hdac1 impairs neuronal differentiation in vitro (Nieto‐Estevez et al. 2022) and the decrease of *Hdac1* expression during early stages of synapse development facilitates excitatory synapse maturation and increases synapse numbers (Akhtar et al. 2009).

## 5 Summary

EVs derived from astrocytes and neurons induce distinct, cell‐specific changes in gene expression. NEVs promote astrocyte proliferation, migration, and metabolic shifts toward glycolysis and lactate production, while AEVs drive neuronal progenitor differentiation, synapse maturation, and upregulate genes involved in synaptic plasticity and memory formation. Most EV‐regulated genes were cell‐type specific, with only one common target.

Transcription factors and RNA binding proteins found in EVs highlight their role in post‐transcriptional regulation and neuronal differentiation. The presence of proteins involved in synaptic activity, neurotransmitter metabolism, calcium homeostasis, and metabolic regulation in COEVs suggests a dynamic adjustment of astrocyte‐neuron interactions in response to physiological demands.

Concluding, our findings presented in this paper underscore the dynamic regulation of cellular processes in astrocytes and neurons by their bi‐directional, EV‐mediated communication.

## 6 Study Limitations

While our results provide new insights into the molecular basis of astrocyte‐neuron communication via EVs, this study has certain limitations. Due to technical constraints, the proteomic analysis of EVs from co‐culture was performed on pooled biological replicates, which precluded differential analysis of obtained results. Additionally, this experiment did not allow for the distinction of proteins derived from astrocytic vesicles from proteins derived from neuronal vesicles.

## Author Contributions

**Daria Hajka:** conceptualization, formal analysis, investigation, methodology, project administration, visualization, writing – original draft. **Paulina Żebrowska‐Różańska:** formal analysis, project administration. **Katarzyna Romańczuk:** investigation. **Jacek R. Wiśniewski:** formal analysis, investigation. **Łukasz Łaczmański:** resources, supervision. **Norbert Łodej:** investigation, methodology. **Krzysztof J. Pawlik:** resources, supervision. **Dariusz Rakus:** conceptualization, funding acquisition, resources. **Agnieszka Gizak:** conceptualization, supervision, writing – original draft, writing – review and editing.

## Funding

This work was supported by Narodowe Centrum Nauki, UMO‐2020/37/B/NZ4/00808.

## Ethics Statement

The protocol used in this work complies with standards of EU Directive 2010/63/EU for animal experiments and was approved by the II Local Scientific Research Ethical Committee, Wroclaw University of Environmental and Life Sciences (permission no WNB.464.2.2020.IR). The study is reported in accordance with ARRIVE guidelines.

## Conflicts of Interest

The authors declare no conflicts of interest.

## Supporting information


**Data S1:** jnc70373‐sup‐0001‐FigureS1‐S5‐TableS1‐S2‐DataS1‐S4.pdf.
**Figure S1:** Size distribution histograms of EVs. (a) A total of 1043 AEVs were measured. The smallest particle detected had a diameter of 15 nm, while the largest reached 233 nm. Only 2.3% of AEVs exceeded 150 nm and were therefore classified as microvesicles, whereas the vast majority (97.7%) fell within the exosomal size range (30–150 nm). The overall mean diameter was 69 nm (SD = 31 nm), with a median of 64 nm and an interquartile range (IQR) of 38 nm. (b) Measurements were performed on 985 NEVs. Particle diameters ranged from 16 to 280 nm. According to the size, 7% of the vesicles were categorized as microvesicles and 93% as exosomes. The mean particle diameter was 65 nm (SD = 39 nm).
**Figure S2:** Internalization of extracellular vesicles. Panel (a) shows astrocyte‐derived EVs (green) internalized by neurons after 1 h of incubation, along with a negative control (fresh culture medium processed in the same way). (b) The panel depicts the uptake of neuron‐derived EVs (green) by astrocytes, together with the negative control. Scale bar = 40 μm. AEVs—astrocytic extracellular vesicles, NEVs, neuronal extracellular vesicles; NA, neuronal monocultures incubated with astrocytic extracellular vesicles; AN, astrocytic monocultures incubated with neuronal extracellular vesicles.
**Figure S3:** Heatmaps of differentially expressed genes (DEGs) in astrocytes and neurons, after incubation with extracellular vesicles (EVs). The heatmaps display the expression patterns of all DEGs in neurons (a) and astrocytes (b) in response to EVs; *n* = 3 (number of independent cell culture preparations for both astrocytes and neurons). Red indicates upregulated gene expression, and blue indicates downregulated expression. *Z*‐score scaling was used to standardize the data. Hierarchical clustering is shown at the top, grouping samples based on similar expression profiles. A, astrocytic monocultures; N, neuronal monocultures; AN, astrocytic monocultures incubated with neuronal extracellular vesicles; NA, neuronal monocultures incubated with astrocytic extracellular vesicles.
**Figure S4:** Cluster analysis of samples. (a) Heatmap of the distance matrix showing pairwise distances between samples; *n* = 3 (number of independent cell culture preparations for both astrocytes and neurons). The color gradient represents distance values, with red indicating smaller distances (higher similarity) and blue indicating larger distances (lower similarity). (b) Dendrogram of hierarchical clustering of the samples, with branches grouped based on their similarity. Distinct clusters are outlined with dashed lines. (c) K‐means clustering plot, ellipses represent the groupings based on k‐means clustering results. Numbers indicate the scores returned by the analysis. A, astrocytic monoculture; N, neuronal monoculture; AN, astrocytic monoculture incubated with neuronal extracellular vesicles; NA, neuronal monoculture incubated with astrocytic extracellular vesicles.
**Figure S5:** (a) Immunofluorescence images of astrocytes stained for Gfap (magenta), a structural protein, to assess cell morphology. Cell nuclei are stained with DAPI (blue). Scale bar = 10 μm. (b) Representative images and quantification of Ki‐67 level in cells' nuclei, a marker of cell proliferation, in astrocytes. The bar plot shows the mean nuclear fluorescence intensity of Ki‐67; *n* = 3 (number of independent cell culture preparations). Results are normalized to the control condition, astrocytes cultured in monoculture (A). The central line represents the median, while the lower and upper edges of the box correspond to the first and third quartiles (Q1 and Q3), respectively. The whiskers indicate the minimum and maximum values within the dataset. Individual points represent biological replicates. Data distribution in both groups was normal (Shapiro–Wilk: A, *W* = 0.97, *p* = 0.38; AN, *W* = 0.97, *p* = 0.28), and homogeneity of variance was confirmed (Levene's test: *F*(1,78) = 1.06, *p* = 0.31). No outlier test was performed. Group differences were assessed using a two‐sided independent samples *t*‐test (*t*(78) = −6.73, *p* = 2.592 × 10^−9^). Scale bar = 40 μm. Error bars show standard deviation (SD). (c) Cell cycle analysis of astrocytes in control and NEVs‐treated condition. The pie chart shows the distribution of cells in G0/G1, S, and G2/M phases for both conditions. A, astrocytic monoculture; AN, astrocytes incubated with neuronal extracellular vesicles for 48 h.
**Table S1:** EV‐associated proteins identified in this study compared with protein content–based EV characterization guidelines from MISEV2023 (Welsh et al. 2024).
**Table S2:** List of mentioned DEGs along with literature reports regarding discussed gene functions.

## Data Availability

The data that support the findings of this study are openly available in NCBI Sequence Read Archive (SRA) at https://www.ncbi.nlm.nih.gov/sra, reference number PRJNA1234574. Supporting Information S1–S4 are available in the RODBUK repository under the DOI: https://doi.org/10.34616/LERXTA.
