# Supplementary material for: Bidirectional Communication Between Astrocytes and Neurons via Extracellular Vesicles: A Multi‐Omics Approach
Source: J Neurochem. 2026 Feb 6;170(2):e70373. doi: 10.1111/jnc.70373 (PMC12879276; doi:10.1111/jnc.70373)
Supplement: Supplementary file 1 — Data S1: jnc70373‐sup‐0001‐FigureS1‐S5‐TableS1‐S2‐DataS1‐S4.pdf. Figure S1: Size distribution histograms of EVs. (a) A total of 1043 AEVs were measured. The smallest particle detected had a diameter of 15 nm, while the largest reached 233 nm. Only 2.3% of AEVs exceeded 150 nm and were therefore classified as microvesicles, whereas the vast majority (97.7%) fell within the exosomal size range (30–150 nm). The overall mean diameter was 69 nm (SD = 31 nm), with a median of 64 nm and an interquartile range (IQR) of 38 nm. (b) Measurements were performed on 985 NEVs. Particle diameters ranged from 16 to 280 nm. According to the size, 7% of the vesicles were categorized as microvesicles and 93% as exosomes. The mean particle diameter was 65 nm (SD = 39 nm). Figure S2: Internalization of extracellular vesicles. Panel (a) shows astrocyte‐derived EVs (green) internalized by neurons after 1 h of incubation, along with a negative control (fresh culture medium processed in the same way). (b) The panel depicts the uptake of neuron‐derived EVs (green) by astrocytes, together with the negative control. Scale bar = 40 μm. AEVs—astrocytic extracellular vesicles, NEVs, neuronal extracellular vesicles; NA, neuronal monocultures incubated with astrocytic extracellular vesicles; AN, astrocytic monocultures incubated with neuronal extracellular vesicles. Figure S3: Heatmaps of differentially expressed genes (DEGs) in astrocytes and neurons, after incubation with extracellular vesicles (EVs). The heatmaps display the expression patterns of all DEGs in neurons (a) and astrocytes (b) in response to EVs; n = 3 (number of independent cell culture preparations for both astrocytes and neurons). Red indicates upregulated gene expression, and blue indicates downregulated expression. Z‐score scaling was used to standardize the data. Hierarchical clustering is shown at the top, grouping samples based on similar expression profiles. A, astrocytic monocultures; N, neuronal monocultures; AN, astrocyti [file JNC-170-0-s001.pdf]

Supplementary information

Bidirectional communication between astrocytes and neurons via extracellular vesicles: A multi-omics approach

Daria Hajka[1], Paulina Żebrowska-Różańska[2], Katarzyna Romańczuk[2], Jacek R. Wiśniewski[3], Łukasz Łaczmański[2], Norbert Łodej[2], Krzysztof J. Pawlik[2], Dariusz Rakus[1], Agnieszka Gizak[1,*]

1 Department of Molecular Physiology and Neurobiology, University of Wrocław, 50-335, Wrocław, Poland

2 Hirszfeld Institute of Immunology and Experimental Therapy, Polish Academy of Sciences, Wrocław, Poland

3 Department of Proteomics and Signal Transduction, Max Planck Institute of Biochemistry, Martinsried, Germany

* agnieszka.gizak@uwr.edu.pl

The current address of Daria Hajka is: Łukasiewicz Research Network - PORT Polish Center for Technology Development, 54-006, Wrocław, Poland.

Supplementary materials and methods

1) Immunofluorescence

Immunofluorescent staining was carried out on paraformaldehyde-fixed cells as previously described (Mamczur et al., 2015). The cells were incubated overnight at 4 °C with respective primary antibodies: rabbit anti-Ki-67 (1:300, Abcam, Cambridge, UK, ab15580, RRID:AB_443209), mouse anti-Gfap (1:1000, Merck KGaA, Darmstadt, Germany, G3893, RRID:AB_477010). The primary antibodies were visualized using the appropriate fluorophore-labeled secondary antibodies: goat anti-rabbit AlexaFluor 488 (1:2000, ThermoFisher Scientific, Waltham, MA, USA, A11034, RRID:AB_2576217), goat anti-mouse AlexaFluor 633 (1:2000, ThermoFisher Scientific, Waltham, MA, USA, A21050, RRID:AB_2535718). Nuclei were stained with DAPI. For immunofluorescence-based experiments cells were incubated with EVs for 48 h.

2) Confocal microscopy

Microscopic observations were performed using the Olympus FV1000 confocal microscope (RRID:SCR_020337), equipped with Plan Apo 60x/1.35 NA oil and UPlanSApo 40x/0.95 NA objectives. The pixel size was set to 0.5 μm, with an exposure time of 2 μs. Fluorescence quantification was carried out using the ImageJ software (RRID:SCR_003070). For Ki-67, which is nuclear proteins, ROIs were established based on DAPI staining.

3) Labeling of extracellular vesicles

To confirm the internalization of extracellular vesicles by target cells, EVs were labeled using the PKH67-FITC dye (Merck KGaA, Darmstadt, Germany, MINI67), according to the manufacturer instruction. After isolation, EVs were resuspended in 500 µL of Diluent C (provided with the dye). Separately, 2 µL of PKH67-FITC was diluted in 500 µL of Diluent C and added to the EV suspension. The mixture was incubated for 5 minutes, after which the labeling reaction was quenched by the addition of 2 mL of 0.5% BSA. The solution was then centrifuged to re-pellet the EVs, and the pellet was added to the culture medium and incubated with the opposing cell type for 1 hour. After fixation, cells were immunostained for cell-specific markers Gfap and Map2, as described above, and subjected to microscopic observations. As a negative control, the labeling procedure was performed on fresh neuronal culture medium (used for all experimental procedures) and no PKH67-FITC signal was detected, confirming the absence of extracellular vesicles in the fresh medium.

4) Flow cytometry

The Guava Muse Cell Analyzer (Luminex, RRID:SCR_020252) was used to determine the distribution of cells across different phases of the cell cycle using the Muse Cell Cycle Kit (Luminex, MCH100106), following the manufacturer's instructions. For this experiment astrocyte were incubated with NEVs for 48 h.

5) Data quantification, presentation, and statistical analysis

For supplementary fluorescence-based experiments, fluorescence intensity was measured using ImageJ with appropriately defined Regions of Interest (ROIs). Each experiment was conducted in three biological replicates, representing independent cell cultures derived from different litters. The mean fluorescence intensity from the control group was normalized to 100% and presented in bar plots. Statistical significance was defined as $p < 0.05$. The specific statistical test used is indicated in the corresponding figure legend. All statistical analyses were performed using R Statistical Software (v4.3.1; R Core Team, 2023).

Supplementary figures

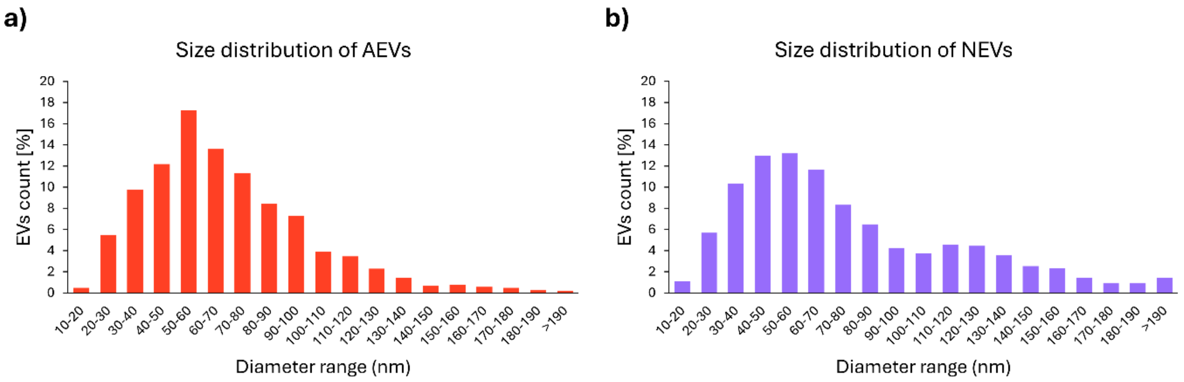

Supplementary Figure 1. Size distribution histograms of EVs. (a) A total of 1,043 AEVs were measured. The smallest particle detected had a diameter of 15 nm, while the largest reached 233 nm. Only 2.3% of AEVs exceeded 150 nm and were therefore classified as microvesicles, whereas the vast majority (97.7%) fell within the exosomal size range (30–150 nm). The overall mean diameter was 69 nm (SD = 31 nm), with a median of 64 nm and an interquartile range (IQR) of 38 nm. (b) Measurements were performed on 985 NEVs. Particle diameters ranged from 16 nm to 280 nm. According to the size, 7% of the vesicles were categorized as microvesicles and 93% as exosomes. The mean particle diameter was 65 nm (SD = 39 nm), with a median of 58 nm and an interquartile range (IQR) of 49 nm.

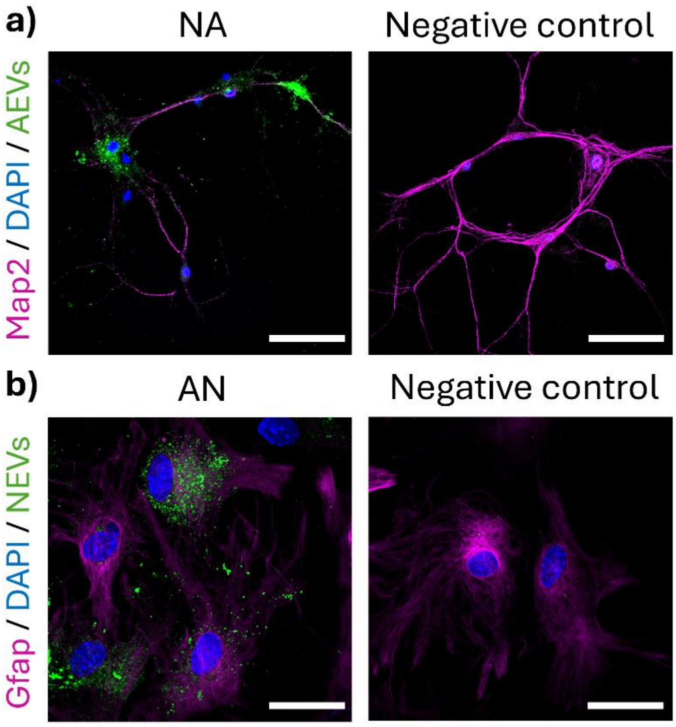

Supplementary Figure 2. Internalization of extracellular vesicles. Panel (a) shows astrocyte-derived EVs (green) internalized by neurons after 1 h of incubation, along with a negative control (fresh culture medium processed in the same way). (b) The panel depicts the uptake of neuron-derived EVs (green) by astrocytes, together with the negative control. Scale bar = 40 µm. AEVs – astrocytic extracellular vesicles, NEVs – neuronal extracellular vesicles, NA – neuronal monocultures incubated with astrocytic extracellular vesicles, AN – astrocytic monocultures incubated with neuronal extracellular vesicles.

a)

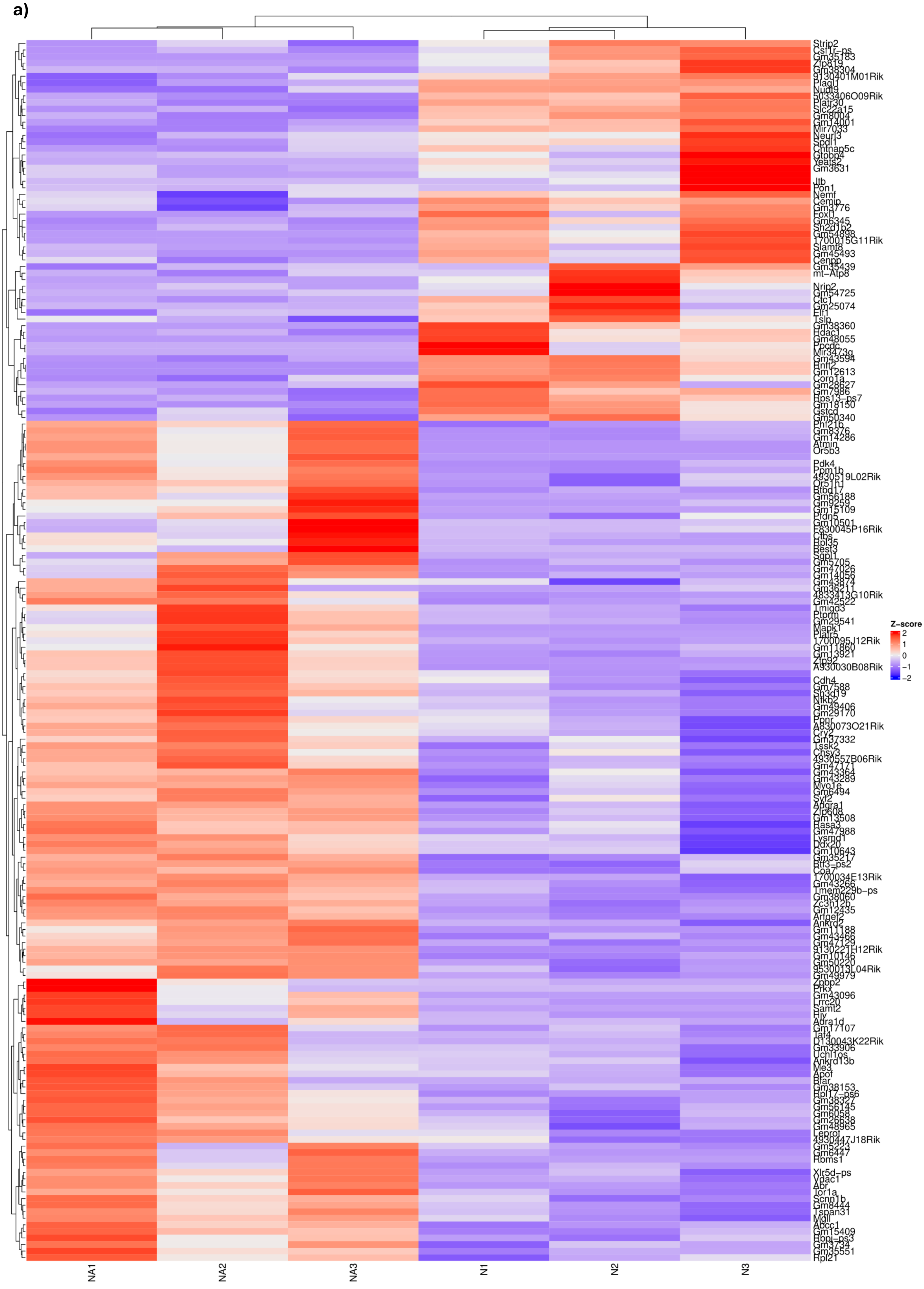
Strip2
Csf1r-ps
Gm35183
Zfp819
Gm38304
9130401M01Rik
Plagl1
Nudt9
5033406O09Rik
Platr30
Slc22a15
Gm8004
Gm14001
Mir7033
Neurl3
Spdl1
Cntnap5c
Gtpbp4
Yeats2
Gm3631
Ltb
Pon1
Nemf
Cemip
Gm3776
Foxl1
Gm6345
Sh2d1b2
Gm54898
1700015G11Rik
Slamf8
Gm45493
Cenpp
Gm35439
mt-Atp8
Nrip2
Gm54725
Ctc1
Gm25074
Elf1
Tslp
Gm38360
Hdac1
Gm48055
Ppcdc
Mir3473g
Gm43594
Rnft1
Gm12613
Coro1a
Gm28627
Gm7986
Rps13-ps7
Gm18150
Gstcd
Gm50340
Phf21b
Gm8376
Gm14286
Atmin
Or5b3
Pdk4
Ppm1b
4930519L02Rik
Or51h1
Btbd17
Gm56188
Gm9259
Gm15109
Prdm5
Gm10501
E830045P16Rik
Cfbs
Rpl35
Best3
Sgpl1
Gm5705
Gm47026
Gm14056
Gm36874
Gm36211
4833413G10Rik
Gm42522
Tmigd3
Ptprm
Gm29541
Mapk1
Platr5
1700095J12Rik
Gm11860
Gm13921
Zfp92
A930030B08Rik
Cdh4
Gm7588
Sh3d19
Nfkb2
Gm49406
Gm29170
Pomt
A830073O21Rik
Cry2
Gm37332
Tssk2
Chsy3
4930557B06Rik
Gm47177
Gm43364
Gm43289
Myo1e
Gm6494
Syt2
Adgra1
Zfp608
Gm13508
Rasa3
Gm47988
Lysmd1
Ddx20
Gm10643
Gm35217
Btf3-ps2
1700034E13Rik
Gm43266
Tmem229b-ps
Gm38060
Zc3h12c
Gm11235
Arfgef2
Ankrd2
Gm11188
9130221H12Rik
Gm10146
Gm50220
9530013L04Rik
Gm49979
Zbbp2
Gm43096
Lrrc20
Samt2
Adra1d
Gm17107
Taf4
D130043K22Rik
Gm33906
Uchl1os
Ankrd13b
Me3
Apof
Gm38153
Rpl17-ps6
Gm38327
Gm56145
Gm6058
Lepr
4930447J18Rik
Gm5223
Gm6447
Rbms1
Xlr5d-ps
Vdac1
Abr
Tor1a
Scnn1b
Gm8444
Tspan31
Mgll
Abcc1
Gm15409
Rbpj-ps3
Gm35551
Rpl21
Z-score
2
1
0
−1
−2
NA1
NA2
NA3
N1
N2
N3

b)

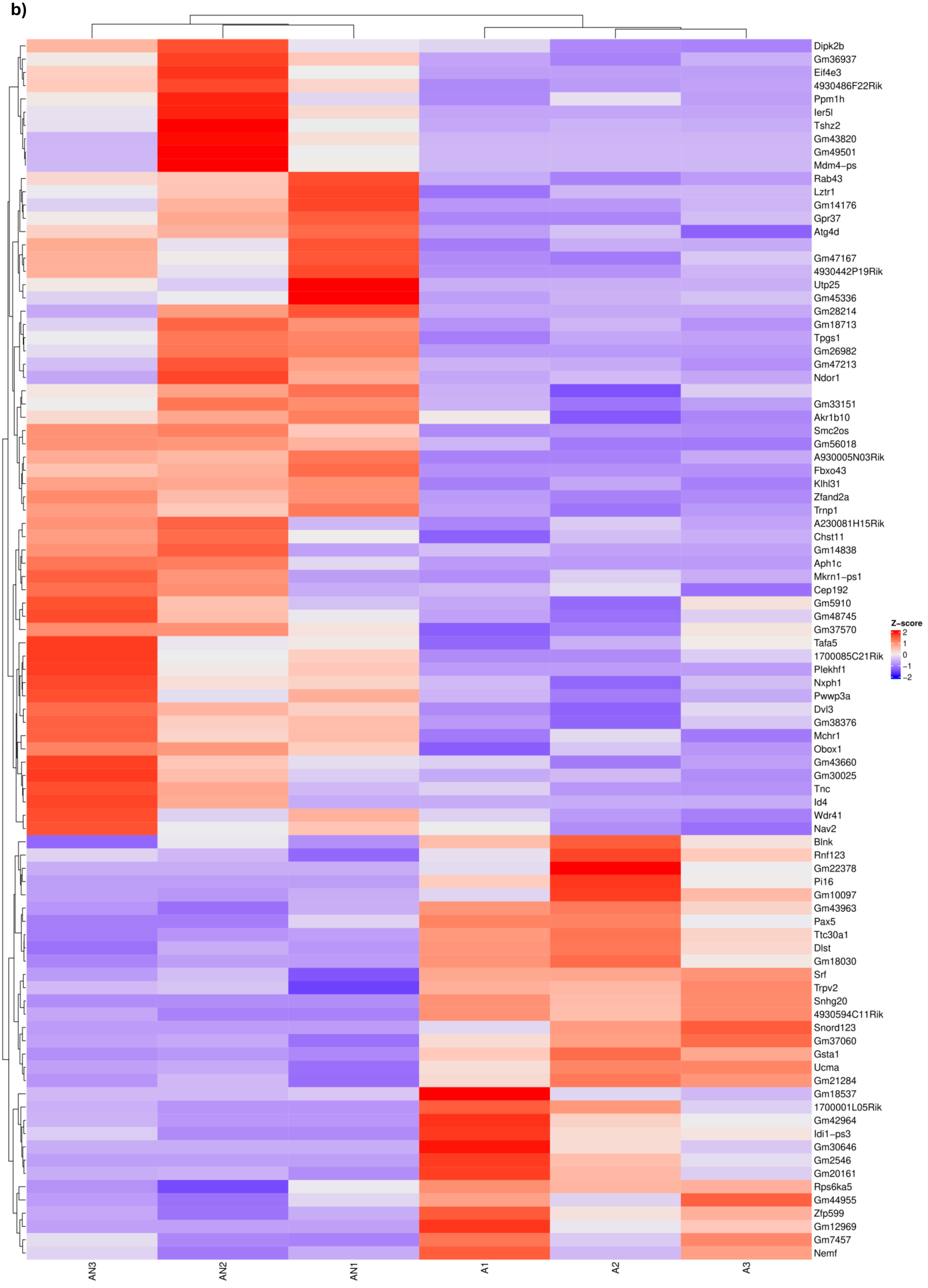

Dipk2b
Gm36937
Eif4e3
4930486F22Rik
Ppm1h
Ier5l
Tshz2
Gm43820
Gm49501
Mdm4−ps
Rab43
Lztr1
Gm14176
Gpr37
Atg4d
Gm47167
4930442P19Rik
Utp25
Gm45336
Gm28214
Gm18713
Tpgs1
Gm26982
Gm47213
Ndor1
Gm33151
Akr1b10
Smc2os
Gm56018
A930005N03Rik
Fbxo43
Klhl31
Zfand2a
Trnp1
A230081H15Rik
Chst11
Gm14838
Aph1c
Mkrn1−ps1
Cep192
Gm5910
Gm48745
Gm37570
Tafa5
1700085C21Rik
Plekhf1
Nxph1
Pwwp3a
Dvl3
Gm38376
Mchr1
Obox1
Gm43660
Gm30025
Tnc
Id4
Wdr41
Nav2
Blnk
Rnf123
Gm22378
Pi16
Gm10097
Gm43963
Pax5
Ttc30a1
Dlst
Gm18030
Srf
Trpv2
Snhg20
4930594C11Rik
Snord123
Gm37060
Gsta1
Ucma
Gm21284
Gm18537
1700001L05Rik
Gm42964
Idi1−ps3
Gm30646
Gm2546
Gm20161
Rps6ka5
Gm44955
Zfp599
Gm12969
Gm7457
Nemf
Z-score
2
1
0
−1
−2
AN3
AN2
AN1
A1
A2
A3

Supplementary Figure 3. Heatmaps of differentially expressed genes (DEGs) in astrocytes and neurons, after incubation with extracellular vesicles (EVs). The heatmaps display the expression patterns of all DEGs in neurons (a) and astrocytes (b) in response to EVs; n = 3 (number of independent cell culture preparations for both astrocytes and neurons). Red indicates upregulated gene expression, and blue indicates downregulated expression. Z-score scaling was used to standardize the data. Hierarchical clustering is shown at the top, grouping samples based on similar expression profiles. A – astrocytic monocultures, N – neuronal monocultures, AN – astrocytic monocultures incubated with neuronal extracellular vesicles, NA – neuronal monocultures incubated with astrocytic extracellular vesicles.

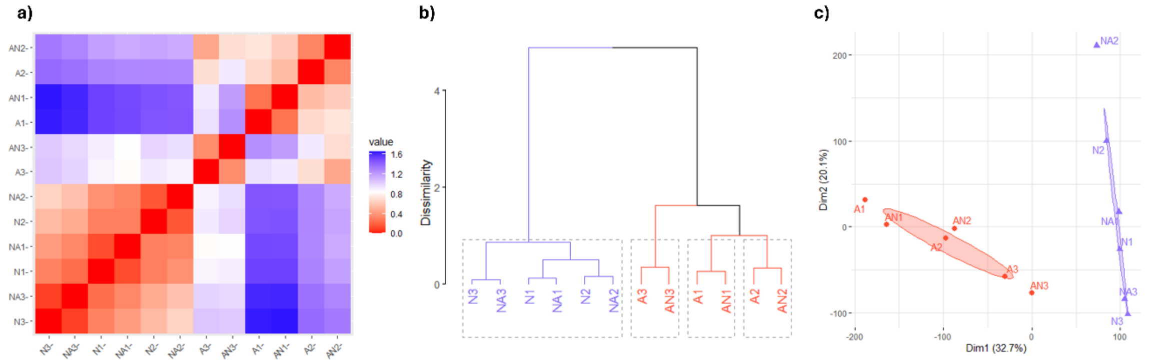

Supplementary Figure 4. Cluster analysis of samples. (a) Heatmap of the distance matrix showing pairwise distances between samples; n = 3 (number of independent cell culture preparations for both astrocytes and neurons). The color gradient represents distance values, with red indicating smaller distances (higher similarity) and blue indicating larger distances (lower similarity). (b) Dendrogram of hierarchical clustering of the samples, with branches grouped based on their similarity. Distinct clusters are outlined with dashed lines. (c) K-means clustering plot, ellipses represent the groupings based on k-means clustering results. Numbers indicate the scores returned by the analysis. A – astrocytic monoculture, N – neuronal monoculture, AN – astrocytic monoculture incubated with neuronal extracellular vesicles, NA – neuronal monoculture incubated with astrocytic extracellular vesicles.

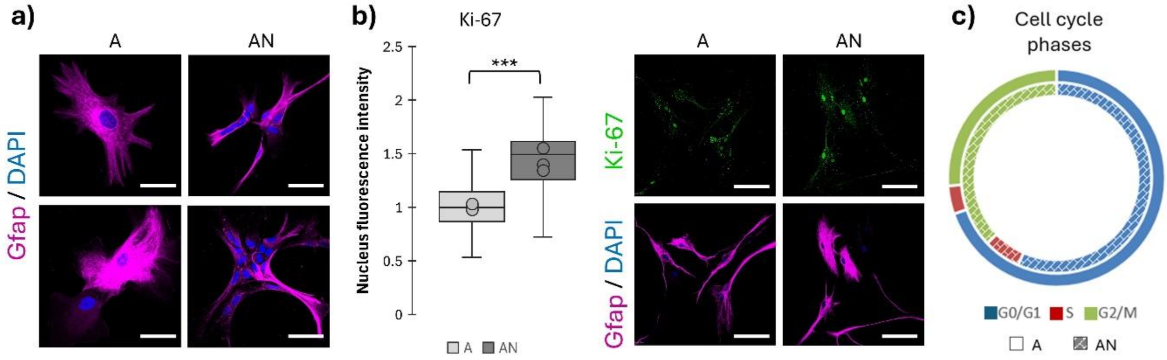

Supplementary Figure 5. (a) Immunofluorescence images of astrocytes stained for Gfap (magenta), a structural protein, to assess cell morphology. Cell nuclei are stained with DAPI (blue). Scale bar = 10 µm. (b) Representative images and quantification of Ki-67 level in cells' nuclei, a marker of cell proliferation, in astrocytes. The bar plot shows the mean nuclear fluorescence intensity of Ki-67; n = 3 (number of independent cell culture preparations). Results are normalized to the control condition, astrocytes cultured in monoculture (A). The central line represents the median, while the lower and upper edges of the box correspond to the first and third quartiles (Q1 and Q3), respectively. The whiskers indicate the minimum and maximum values within the dataset. Individual points represent biological replicates. Data distribution in both groups was normal (Shapiro–Wilk: A, $W = 0.97$, $p = 0.38$; AN, $W = 0.97$, $p = 0.28$), and homogeneity of variance was confirmed (Levene's test: $F(1,78) = 1.06$, $p = 0.31$). No outlier test was performed. Group differences were assessed using a two-sided independent samples t-test ($t(78) = -6.73$, $p = 2.592 \times 10^{-9}$). Scale bar = 40 µm. Error bars show standard deviation (SD). (c) Cell cycle

analysis of astrocytes in control and NEVs-treated condition. The pie chart shows the distribution of cells in G0/G1, S, and G2/M phases for both conditions. A – astrocytic monoculture, AN – astrocytes incubated with neuronal extracellular vesicles for 48 h.

Supplementary Tables

Supplementary Table 1. EV-associated proteins identified in this study compared with protein content–based EV characterization guidelines from MISEV2023 (Welsh et al., 2024)

	c (pmol/mg) AEVs	c (pmol/mg) COEVs	c (pmol/mg) NEVs
1- Transmembrane			
**1a: multi-pass transmembrane proteins**			
CD9	3.15E+01	0.00E+00	0.00E+00
CD63	3.96E+01	0.00E+00	0.00E+00
CD81	1.89E+02	4.79E+01	5.50E+01
CD82	-	-	-
CD47	-	-	-
TSAP6	-	-	-
**1b: single-pass transmembrane proteins**			
Itga3	1.04E+01	0.00E+00	0.00E+00
Itga6	0.00E+00	3.54E+00	5.16E+00
Itga7	1.74E+01	0.00E+00	0.00E+00
Itgav	0.00E+00	2.22E+00	0.00E+00
TFR2	-	-	-
LAMP1	0.00E+00	0.00E+00	4.29E+00
LAMP2	-	-	-
SDC	-	-	-
BSG	7.49E+01	1.64E+01	3.07E+01
ADAM10	-	-	-
**1c: GPI- or lipid-anchored proteins**			
GPC1 (4,6)	0.00E+00	8.93E-01	0.00E+00
CD73(NT5E)	-	-	-
SCD59	-	-	-
2- Cytosolic proteins in EVs			
**2a: with lipid or membrane protein-binding ability**			
TSG101	0.00E+00	8.03E+00	7.06E+00
CHMP(4b)	1.96E+01	2.10E+01	1.74E+01
ALIX (PDCD6IP)	1.74E+01	1.39E+00	0.00E+00
Vps28	3.08E+01	0.00E+00	1.34E+01
Vps35	0.00E+00	5.76E-01	2.83E+00
ARRDC1	-	-	-
FLOT1	-	-	-
FLOT2	-	-	-
CAV	-	-	-
SDCBP	2.17E+01	0.00E+00	6.11E+00

**2b: promiscuous incorporation into EVs (and possibly NVEPs)**			
HSC70 (HSPA8)	1.64E+02	2.24E+02	1.28E+02
HSP84 (HSP90AB1)	5.42E+01	1.34E+02	1.70E+02
Actb	0.00E+00	1.32E+01	2.58E+01
Actbl2	0.00E+00	4.70E+00	0.00E+00
Actc1; Acta2; Actg2; Acta1	4.97E+02	3.30E+02	4.05E+02
Actg1	2.41E+03	4.06E+03	1.68E+03
Tuba1b; Tuba1a; Tuba1c; Tuba3a; Tuba4a	1.52E+02	4.04E+02	5.46E+02
Tubb1	7.51E+00	5.78E+00	0.00E+00
Tubb2b; Tubb2a	3.46E+01	5.47E+02	4.80E+02
Tubb4b; Tubb4a	1.17E+01	7.53E+01	6.85E+01
Tubb5	5.26E+00	2.23E+01	7.90E+01
Tubb6	0.00E+00	3.44E+00	5.51E+00
GAPDH	3.61E+02	8.60E+01	5.26E+02
3- Major components of non-EV co-isolated structures (NVEPs)			
**3a: lipoproteins**			
lipoproteins	-	-	-
Apoe	5.28E+02	8.18E+02	1.90E+03
**3b: protein and protein/nucleic acid aggregates**			
Igsf8	0.00E+00	2.66E+00	1.59E+01
Tamm-Horsfall protein	-	-	-
Uromodulin	-	-	-
UMOD	-	-	-
urine	-	-	-
albumin	-	-	-
YWAH	-	-	-
AGO	-	-	-
**3c: exomere or supermere-enriched components**			
HSP90AA	0.00E+00	9.63E+00	7.50E+00
HSP90B	0.00E+00	0.00E+00	1.63E+01
TGFBI	-	-	-
HSPA13	-	-	-
LDHA	0.00E+00	1.15E+02	1.04E+02
LDHB	8.11E+00	2.20E+01	7.75E+01

Supplementary Table 2. List of mentioned DEGs along with literature reports regarding discussed gene functions

Gene	NGS result	Literature reports
Abcc1	Upregulation (NA)	Overexpression of *Abcc1* promotes breast cancer cell proliferation and migration and enhances angiogenesis and lymphangiogenesis in endothelial cells (Yamada et al., 2018).
Apof	Upregulation (NA)	Apof is a sialoglycoprotein found in the HDL and LDL fractions of human serum. Expression of brain apolipoproteins is significantly altered in neurological and psychiatric disorders (Elliott et al., 2010).
Atg4d	Upregulation (AN)	*Atg4d* is important during cellular stress and differentiation, it regulates mitochondrial physiology, ROS production, mitophagy, and cell viability (Betin et al., 2012).
Atmin	Upregulation (NA)	Mouse embryos lacking *Atmin* (atminΔ/Δ) exhibited in utero lethality and an increased number of cells positive for phosphorylated histone H2AX, indicating elevated DNA damage (Kanu et al., 2010).
Bfar	Upregulation (NA)	Overexpression of *Bfar* in immortalized rat neuronal cells provides significant protection against various cell death stimuli, including those activating mitochondrial, TNF-family death receptor, and ER stress-related apoptotic pathways. In contrast, *Bfar* downregulation via antisense oligonucleotides sensitizes neuronal cells to apoptosis (Roth et al., 2003).
Cep192	Upregulation (AN)	Cep192, a key centrosomal protein, supports mitotic spindle assembly and function in mammalian cells, its depletion impairs cell motility and disrupts normal cell polarization (O'Rourke et al., 2014).
Coa7	Upregulation (NA)	Coa7 plays a role in assembling mitochondrial respiratory chain complexes involved in oxidative phosphorylation. Loss-of-function mutations in *Coa7* are responsible for the phenotype observed in the patients with spinocerebellar ataxia (Higuchi et al., 2018).
Ctc1	Downregulation (NA)	Ctc1-Stn1-Ten1 (CST) complex plays a crucial role in countering replication stress and protecting the stability of genomic fragile sites. *Ctc1* mutations impair cell proliferation under unstressed conditions and significantly reduce clonal viability under replication stress (Wang & Chai, 2018).
Dlst	Downregulation (AN)	Dlst is a component of the α-ketoglutarate dehydrogenase complex, which regulates the entry of glutamine into the tricarboxylic acid (TCA) cycle for oxidative decarboxylation. Elevated expression of *Dlst* predicts poor treatment outcomes and aggressive disease in neuroblastoma patients (Anderson et al., 2021).
Gpr37	Upregulation (AN)	Loss of *Gpr37L1* in mice does not affect astrocyte or neuronal input resistance or resting potential but inhibits astrocyte glutamate transporters and reduces neuronal NMDAR activity during ischemia (Jolly et al., 2018).
Hdac1	Downregulation (NA)	A decrease in the activity of Hdac1 during early synaptic development strongly enhances excitatory synapse maturation and modestly increases synapse numbers (Akhtar et al., 2009).
Id4	Upregulation (AN)	*Id4* overexpression enhances astrocyte proliferation in the CA1 layer of the injured hippocampus until 3 days post-lesion. *Id4* in promoting astrocyte activation following excitotoxin-induced neuronal death in the hippocampus (Lee et al., 2011).
Mapk1	Upregulation (NA)	MAPK families are crucial in regulating proliferation, differentiation, development, transformation, and apoptosis. The *Mapk1* gene shows an up-down expression pattern across the lifespan and all 11 cortical regions, being upregulated during postnatal development and downregulated with aging (Qiu et al., 2021).
Mgll	Upregulation (NA)	Stable knockdown of *Mgll* suppresses tumor proliferation and metastasis both in vitro and in vivo, mechanistically, *Mgll* regulates Cyclin D1 and Cyclin B1 in lung adenocarcinoma cells (Zhang et al., 2020).
Mt-Atp8	Downregulation (NA)	Mt-Atp8 is a part of the mitochondrial proton-transporting ATP synthase complex. Point mutation in *Mt-Atp8* in a child caused early-onset ataxia, psychomotor delay, and microcephaly (Fragaki et al., 2019).

Ndor1	Upregulation (AN)	Ndor1 catalyzes the transfer of electrons from NADPH through flavin mononucleotide (FMN) and flavin adenine dinucleotide (FAD) cofactors to potential redox partners. The Ndor1-anamorsin interaction is proposed to play a role in regulating cell survival and death mechanisms (Banci et al., 2013).
Nemf	Downregulation (NA)<br>Downregulation (AN)	*Nemf* modifies translation products of nonstop mRNAs, which are major erroneous mRNAs in mammals. Overproduction of nonstop mRNAs leads to polypeptide chains aggregation, caspase-3-dependent apoptosis, and impaired neuronal morphogenesis, all of which are alleviated by *Nemf* depletion (Udagawa et al., 2021).
Nfkb2	Upregulation (NA)	In a forebrain neuronal NF-κB-deficient mouse model, memory formation, synaptic transmission, and plasticity was impaired (Kaltschmidt et al., 2006).
Nudt9	Downregulation (NA)	Nudt9 enzyme from the Nudix hydrolase family, is an evolutionarily conserved mitochondrial ADP-ribose pyrophosphatase that breaks down ADP-ribose (ADPR) into AMP and ribose 5'-phosphate (Perraud et al., 2003).
Pdk4	Upregulation (NA)	The brain derives most of its energy from the oxygen-dependent metabolism of glucose, with the mitochondrial *Pdk4* playing a crucial regulatory role in glucose oxidation. Changes in PDKs and neuron-astroglia metabolic interactions are probably linked to the development of various neurological disorders (Kumar Jha et al., 2012).
Pfdn5	Upregulation (NA)	In transgenic mice model of Alzheimer's with impaired synaptic plasticity Pfdn5 mRNA and protein levels in the hippocampus were significantly reduced in an age-dependent manner (Kadoyama et al., 2019).
Phf21b	Upregulation (NA)	The depletion of *Phf21b* impairs neuronal differentiation, speeds up progenitor cell cycles, and causes their retention in the proliferative zones of the cortex (Basu et al., 2020).
Plag1	Downregulation (NA)	The loss of *Plag1* diminishes progenitor proliferation and limits their generation in the neocortex in vivo (Gasperoni et al., 2024).
Plekhf1	Upregulation (AN)	Increased expression of *Plekhf1* causes the formation of enlarged endosomes. *Plekhf1* also modulates the membrane density of certain receptors and participates in endocytosis and autophagy processes (W. J. Lin et al., 2012).
Pon1	Downregulation (NA)	*Pon1* knockout mice show increased lipid peroxidation and macrophage oxidative stress (Rozenberg et al., 2003), along with elevated aortic superoxide anion levels and leukocyte adhesion (Ng et al., 2008).
Ppcdc	Downregulation (NA)	Patient-derived fibroblasts with point mutation in *Ppcdc* gene show reduction in CoA levels. The cells demonstrated clear energy deficiencies, with defects in mitochondrial respiration and reliance primarily on glycolytic ATP synthesis (Bravo-Alonso et al., 2023).
Ppm1b	Upregulation (NA)	Ppm1b serves as a key negative regulator of stress signaling, directly dephosphorylating upstream proteins to inhibit the activation of p38 and JNK pathways. Depletion of *Ppm1b* in osteosarcoma cell line suppresses proliferation (Miller et al., 2018).
Ptprm	Upregulation (NA)	*Ptprm* regulates synapse formation in a zinc-dependent manner (Mo et al., 2022).
Pwwp3a	Upregulation (AN)	Pwwp3a is identified as a chromatin architectural component that supports cell survival in response to DNA damage. Depletion of *Pwwp3a* leads to chromatin compaction (Huen et al., 2010).
Rbms1	Upregulation (NA)	The loss of *Rbms1* disrupts radial migration, neuronal progenitor polarization, and their differentiation into mature neurons during the development of the neocortex (Habib et al., 2022).
Rnft2	Downregulation (NA)	Rnft2 negatively regulates IL-3-dependent cellular responses by promoting IL-3Rα ubiquitination and degradation in the proteasome. The RNTF2/IL-3Rα/IL-3 axis may play a role in regulating innate immune responses in the lung (Tong et al., 2020).
Sgpl1	Upregulation (NA)	Constitutive ablation of *Sgpl1* in the brain leads to altered presynaptic architecture, including a significant decrease in the number and density of

		synaptic vesicles and impaired synaptic short-term plasticity in hippocampal neurons (Mitroi et al., 2016).
Sh2d1b2	Downregulation (NA)	*Sh2d1b2* regulates the effector functions of natural killer (NK) cells by modulating signal transduction through Cd244/2B4. It acts as an inhibitor of Cd244-mediated NK cell functions, including cytotoxicity and IFN-γ production (Roncagalli et al., 2005).
Slamf8	Downregulation (NA)	Knockdown of *Slamf8* reduces Hypoxia/Reoxygenation-induced cardiomyocyte death, ferroptosis, and oxidative stress (Zheng et al., 2024).
Srf	Downregulation (AN)	Conditional deletion of *Srf* in astrocytes triggers astrogliosis, characterized by hypertrophic morphology and elevated expression of Gfap, vimentin, and nestin (Jain et al., 2021).
Tnc	Upregulation (AN)	The loss of *Tnc* results in prolonged generation and delayed migration of immature astrocytes in vivo (Karus et al., 2011).
Tor1a	Upregulation (NA)	Loss of *Tor1a* in mouse embryos disrupts neuronal migration, while the overall neurogenesis process remains unaffected (McCarthy et al., 2012).
Trnp1	Upregulation (AN)	Elevated *Trnp1* levels drive neural stem cell self-renewal and tangential expansion, while reduced levels promote radial expansion, significantly increasing intermediate progenitors and basal radial glial cells (Stahl et al., 2013).
Tslp	Downregulation (NA)	Epithelial-derived Tslp controls ROS production and mitophagy via AMPK activation and histone modification, and influences M1/M2 chemokine expression in human monocytes (Y. C. Lin et al., 2022).
Vdac1	Upregulation (NA)	Vdac1 participates in mitochondria-mediated apoptosis by regulating the release of apoptotic proteins and interacting with anti-apoptotic proteins (Shoshan-Barmatz et al., 2017).
Wdr41	Upregulation (AN)	The SMCR8-WDR41-C9ORF72 complex functions as a regulator of autophagy and lysosomal activity. Homozygous *Wdr41* mutant mice exhibit hyperactivation of T cells and elevated basal serum IL-12p40 levels (McAlpine et al., 2018).

AN – changes in gene expression observed in astrocytic monoculture incubated with neuronal extracellular vesicles, NA – changes in gene expression observed in neuronal monoculture incubated with astrocytic extracellular vesicles.

List of Supplementary Data Files:

Supplementary Data 1 - proteomics quantitative data.xls

Supplementary Data 2 - proteomics functional analysis.xls

Supplementary Data 3 - transcriptome DEGs.xls

Supplementary Data 4 - transcriptome functional analysis.xls

All data files are available under a single DOI at the RODBUK repository: https://doi.org/10.34616/LERXTA

References

Akhtar, M. W., Raingo, J., Nelson, E. D., Montgomery, R. L., Olson, E. N., Kavalali, E. T., & Monteggia, L. M. (2009). Histone deacetylases 1 and 2 form a developmental switch that controls excitatory synapse maturation and function. *The Journal of Neuroscience : The Official Journal of the Society for Neuroscience*, *29*(25), 8288–8297. https://doi.org/10.1523/JNEUROSCI.0097-09.2009

Anderson, N. M., Qin, X., Finan, J. M., Lam, A., Athoe, J., Missiaen, R., Skuli, N., Kennedy, A., Saini, A. S., Tao, T., Zhu, S., Nissim, I., Look, A. T., Qing, G., Simon, M. C., & Feng, H. (2021). Metabolic Enzyme DLST Promotes Tumor Aggression and Reveals a Vulnerability to OXPHOS Inhibition in High-Risk Neuroblastoma. *Cancer Research*, *81*(17), 4417–4430. https://doi.org/10.1158/0008-5472.CAN-20-2153

Banci, L., Bertini, I., Calderone, V., Ciofi-Baffoni, S., Giachetti, A., Jaiswal, D., Mikolajczyk, M., Piccioli, M., & Winkelmann, J. (2013). Molecular view of an electron transfer process essential for iron-sulfur protein biogenesis. *Proceedings of the National Academy of Sciences of the United States of America*, *110*(18), 7136–7141. https://doi.org/10.1073/PNAS.1302378110

Basu, A., Mestres, I., Sahu, S. K., Tiwari, N., Khongwir, B., Baumgart, J., Singh, A., Calegari, F., & Tiwari, V. K. (2020). Phf21b imprints the spatiotemporal epigenetic switch essential for neural stem cell differentiation. *Genes & Development*, *34*(17–18), 1190–1209. https://doi.org/10.1101/GAD.333906.119

Betin, V. M. S., MacVicar, T. D. B., Parsons, S. F., Anstee, D. J., & Lane, J. D. (2012). A cryptic mitochondrial targeting motif in Atg4D links caspase cleavage with mitochondrial import and oxidative stress. *Autophagy*, *8*(4), 664–676. https://doi.org/10.4161/AUTO.19227

Bravo-Alonso, I., Morin, M., Arribas-Carreira, L., Álvarez, M., Pedrón-Giner, C., Soletto, L., Santolaria, C., Ramón-Maiques, S., Ugarte, M., Rodríguez-Pombo, P., Ariño, J., Moreno-Pelayo, M. Á., & Pérez, B. (2023). Pathogenic variants of the coenzyme A biosynthesis-associated enzyme phosphopantothenoylcysteine decarboxylase cause autosomal-recessive dilated cardiomyopathy. *Journal of Inherited Metabolic Disease*, *46*(2), 261–272. https://doi.org/10.1002/JIMD.12584

Elliott, D. A., Weickert, C. S., & Garner, B. (2010). Apolipoproteins in the brain: implications for neurological and psychiatric disorders. *Clinical Lipidology*, *51*(4), 555–573. https://doi.org/10.2217/CLP.10.37

Fragaki, K., Chaussenot, A., Serre, V., Acquaviva, C., Bannwarth, S., Rouzier, C., Chabrol, B., & Paquis-Flucklinger, V. (2019). A novel variant m.8561C>T in the overlapping region of MT-ATP6 and MT-ATP8 in a child with early-onset severe neurological signs. *Molecular Genetics and Metabolism Reports*, *21*. https://doi.org/10.1016/J.YMGMR.2019.100543

Gasperoni, J. G., Tran, S. C., Grommen, S. V. H., De Groef, B., & Dworkin, S. (2024). The Role of PLAG1 in Mouse Brain Development and Neurogenesis. *Molecular Neurobiology*, *61*(8), 5851–5867. https://doi.org/10.1007/S12035-024-03943-W

Habib, K., Bishayee, K., Kang, J., Sadra, A., & Huh, S. O. (2022). RNA Binding Protein Rbms1 Enables Neuronal Differentiation and Radial Migration during Neocortical Development by Binding and Stabilizing the RNA Message for Efr3a. *Molecules and Cells*, *45*(8), 588–602. https://doi.org/10.14348/MOLCELLS.2022.0044

Higuchi, Y., Okunushi, R., Hara, T., Hashiguchi, A., Yuan, J., Yoshimura, A., Murayama, K., Ohtake, A., Ando, M., Hiramatsu, Y., Ishihara, S., Tanabe, H., Okamoto, Y., Matsuura, E., Ueda, T., Toda, T., Yamashita, S., Yamada, K., Koide, T., … Takashima, H. (2018). Mutations in COA7 cause spinocerebellar ataxia with axonal neuropathy. *Brain : A Journal of Neurology*, *141*(6), 1622–1636. https://doi.org/10.1093/BRAIN/AWY104

Huen, M. S. Y., Huang, J., Leung, J. W. C., Sy, S. M. H., Leung, K. M., Ching, Y. P., Tsao, S. W., & Chen, J. (2010). Regulation of chromatin architecture by the PWWP domain-containing DNA damage-responsive factor EXPAND1/MUM1. *Molecular Cell*, *37*(6), 854–864. https://doi.org/10.1016/J.MOLCEL.2009.12.040

Jain, M., Das, S., Lu, P. P. Y., Virmani, G., Soman, S., Thumu, S. C. R., Gutmann, D. H., & Ramanan, N. (2021). SRF Is Required for Maintenance of Astrocytes in Non-Reactive State in the Mammalian Brain. *ENeuro*, *8*(1), 1–15. https://doi.org/10.1523/ENEURO.0447-19.2020

Jolly, S., Bazargani, N., Quiroga, A. C., Pringle, N. P., Attwell, D., Richardson, W. D., & Li, H. (2018). G protein-coupled receptor 37-like 1 modulates astrocyte glutamate transporters and neuronal NMDA receptors and is neuroprotective in ischemia. *Glia*, *66*(1), 47–61. https://doi.org/10.1002/GLIA.23198

Kadoyama, K., Matsuura, K., Takano, M., Maekura, K., Inoue, Y., & Matsuyama, S. (2019). Changes in the expression of prefoldin subunit 5 depending on synaptic plasticity in the mouse hippocampus. *Neuroscience Letters*, *712*. https://doi.org/10.1016/J.NEULET.2019.134484

Kaltschmidt, B., Ndiaye, D., Korte, M., Pothion, S., Arbibe, L., Prüllage, M., Pfeiffer, J., Lindecke, A., Staiger, V., Israël, A., Kaltschmidt, C., & Mémet, S. (2006). NF-kappaB regulates spatial memory formation and synaptic plasticity through protein kinase A/CREB signaling. *Molecular and Cellular Biology*, *26*(8), 2936–2946. https://doi.org/10.1128/MCB.26.8.2936-2946.2006

Kanu, N., Penicud, K., Hristova, M., Wong, B., Irvine, E., Plattner, F., Raivich, G., & Behrens, A. (2010). The ATM cofactor ATMIN protects against oxidative stress and accumulation of DNA damage in the aging brain. *The Journal of Biological Chemistry*, *285*(49), 38534–38542. https://doi.org/10.1074/JBC.M110.145896

Karus, M., Denecke, B., Ffrench-Constant, C., Wiese, S., & Faissner, A. (2011). The extracellular matrix molecule tenascin C modulates expression levels and territories of key patterning genes during spinal cord astrocyte specification. *Development (Cambridge, England)*, *138*(24), 5321–5331. https://doi.org/10.1242/DEV.067413

Kumar Jha, M., Jeon, S., & Suk, K. (2012). Pyruvate Dehydrogenase Kinases in the Nervous System: Their Principal Functions in Neuronal-glial Metabolic Interaction and Neuro-metabolic Disorders. *Current Neuropharmacology*, *10*(4), 393–403. https://doi.org/10.2174/157015912804143586

Lee, Y. S., Kang, J. W., Lee, Y. H., & Kim, D. W. (2011). ID4 mediates proliferation of astrocytes after excitotoxic damage in the mouse hippocampus. *Anatomy & Cell Biology*, *44*(2), 128. https://doi.org/10.5115/ACB.2011.44.2.128

Lin, W. J., Yang, C. Y., Li, L. L., Yi, Y. H., Chen, K. W., Lin, Y. C., Liu, C. C., & Lin, C. H. (2012). Lysosomal targeting of phafin1 mediated by Rab7 induces autophagosome formation. *Biochemical and Biophysical Research Communications*, *417*(1), 35–42. https://doi.org/10.1016/J.BBRC.2011.11.043

Lin, Y. C., Lin, Y. C., Tsai, M. L., Liao, W. T., & Hung, C. H. (2022). TSLP regulates mitochondrial ROS-induced mitophagy via histone modification in human monocytes. *Cell & Bioscience*, *12*(1). https://doi.org/10.1186/S13578-022-00767-W

Mamczur, P., Borsuk, B., Paszko, J., Sas, Z., Mozrzymas, J., Wiśniewski, J. R., Gizak, A., & Rakus, D. (2015). Astrocyte-neuron crosstalk regulates the expression and subcellular localization of carbohydrate metabolism enzymes. *Glia*, *63*(2), 328–340. https://doi.org/10.1002/GLIA.22753

McAlpine, W., Sun, L., Wang, K. wen, Liu, A., Jain, R., Miguel, M. S., Wang, J., Zhang, Z., Hayse, B., McAlpine, S. G., Choi, J. H., Zhong, X., Ludwig, S., Russell, J., Zhan, X., Choi, M., Li, X., Tang, M., Moresco, E. M. Y., … Turer, E. (2018). Excessive endosomal TLR signaling causes inflammatory disease in mice with defective SMCR8-WDR41-C9ORF72 complex function. *Proceedings of the National Academy of Sciences of the United States of America*, *115*(49), E11523–E11531. https://doi.org/10.1073/PNAS.1814753115

McCarthy, D. M., Gioioso, V., Zhang, X., Sharma, N., & Bhide, P. G. (2012). Neurogenesis and neuronal migration in the forebrain of the TorsinA knockout mouse embryo. *Developmental Neuroscience*, *34*(4), 366–378. https://doi.org/10.1159/000342260

Miller, R. E., Uwamahoro, N., & Park, J. H. (2018). PPM1B depletion in U2OS cells supresses cell growth through RB1-E2F1 pathway and stimulates bleomycin-induced cell death. *Biochemical and Biophysical Research Communications*, *500*(2), 391–397. https://doi.org/10.1016/J.BBRC.2018.04.084

Mitroi, D. N., Deutschmann, A. U., Raucamp, M., Karunakaran, I., Glebov, K., Hans, M., Walter, J., Saba, J., Gräler, M., Ehninger, D., Sopova, E., Shupliakov, O., Swandulla, D., & Van Echten-Deckert, G. (2016). Sphingosine 1-phosphate lyase ablation disrupts presynaptic architecture and function via an ubiquitin- proteasome mediated mechanism. *Scientific Reports*, *6*. https://doi.org/10.1038/SREP37064

Mo, X., Liu, M., Gong, J., Mei, Y., Chen, H., Mo, H., Yang, X., & Li, J. (2022). PTPRM Is Critical for Synapse Formation Regulated by Zinc Ion. *Frontiers in Molecular Neuroscience*, *15*. https://doi.org/10.3389/FNMOL.2022.822458

Ng, D. S., Chu, T., Esposito, B., Hui, P., Connelly, P. W., & Gross, P. L. (2008). Paraoxonase-1 deficiency in mice predisposes to vascular inflammation, oxidative stress, and thrombogenicity in the absence of hyperlipidemia. *Cardiovascular Pathology : The Official Journal of the Society for Cardiovascular Pathology*, *17*(4), 226–232. https://doi.org/10.1016/J.CARPATH.2007.10.001

O'Rourke, B. P., Gomez-Ferreria, M. A., Berk, R. H., Hackl, A. M. U., Nicholas, M. P., O'Rourke, S. C., Pelletier, L., & Sharp, D. J. (2014). Cep192 controls the balance of centrosome and non-centrosomal microtubules during interphase. *PloS One*, *9*(6). https://doi.org/10.1371/JOURNAL.PONE.0101001

Perraud, A. L., Shen, B., Dunn, C. A., Rippe, K., Smith, M. K., Bessman, M. J., Stoddard, B. L., & Scharenberg, A. M. (2003). NUDT9, a member of the Nudix hydrolase family, is an evolutionarily conserved mitochondrial ADP-ribose pyrophosphatase. *The Journal of Biological Chemistry*, *278*(3), 1794–1801. https://doi.org/10.1074/JBC.M205601200

Qiu, A., Zhang, H., Kennedy, B. K., & Lee, A. (2021). Spatio-temporal correlates of gene expression and cortical morphology across lifespan and aging. *NeuroImage*, *224*. https://doi.org/10.1016/J.NEUROIMAGE.2020.117426

Roncagalli, R., Taylor, J. E. R., Zhang, S., Shi, X., Chen, R., Cruz-Munoz, M. E., Yin, L., Latour, S., & Veillette, A. (2005). Negative regulation of natural killer cell function by EAT-2, a SAP-related adaptor. *Nature Immunology*, *6*(10), 1002–1010. https://doi.org/10.1038/NI1242

Roth, W., Kermer, P., Krajewska, M., Welsh, K., Davis, S., Krajewski, S., & Reed, J. C. (2003). Bifunctional apoptosis inhibitor (BAR) protects neurons from diverse cell death pathways. *Cell Death and Differentiation*, *10*(10), 1178–1187. https://doi.org/10.1038/SJ.CDD.4401287

Rozenberg, O., Rosenblat, M., Coleman, R., Shih, D. M., & Aviram, M. (2003). Paraoxonase (PON1) deficiency is associated with increased macrophage oxidative stress: Studies in PON1-knockout mice. *Free Radical Biology and Medicine*, *34*(6), 774–784. https://doi.org/10.1016/S0891-5849(02)01429-6

Shoshan-Barmatz, V., Maldonado, E. N., & Krelin, Y. (2017). VDAC1 at the crossroads of cell metabolism, apoptosis and cell stress. *Cell Stress*, *1*(1), 11–36. https://doi.org/10.15698/CST2017.10.104

Stahl, R., Walcher, T., De Juan Romero, C., Pilz, G. A., Cappello, S., Irmler, M., Sanz-Aquela, J. M., Beckers, J., Blum, R., Borrell, V., & Götz, M. (2013). Trnp1 regulates expansion and folding of the mammalian cerebral cortex by control of radial glial fate. *Cell*, *153*(3), 535–549. https://doi.org/10.1016/J.CELL.2013.03.027

Tong, Y., Lear, T. B., Evankovich, J., Chen, Y., Londino, J. D., Myerburg, M. M., Zhang, Y., Popescu, I. D., McDyer, J. F., McVerry, B. J., Lockwood, K. C., Jurczak, M. J., Liu, Y., & Chen, B. B. (2020). The RNFT2/IL-3Rα axis regulates IL-3 signaling and innate immunity. *JCI Insight*, *5*(3). https://doi.org/10.1172/JCI.INSIGHT.133652

Udagawa, T., Seki, M., Okuyama, T., Adachi, S., Natsume, T., Noguchi, T., Matsuzawa, A., & Inada, T. (2021). Failure to Degrade CAT-Tailed Proteins Disrupts Neuronal Morphogenesis and Cell Survival. *Cell Reports*, *34*(1). https://doi.org/10.1016/J.CELREP.2020.108599

Wang, Y., & Chai, W. (2018). Pathogenic CTC1 mutations cause global genome instabilities under replication stress. *Nucleic Acids Research*, *46*(8), 3981–3992. https://doi.org/10.1093/NAR/GKY114

Yamada, A., Nagahashi, M., Aoyagi, T., Huang, W. C., Lima, S., Hait, N. C., Maiti, A., Kida, K., Terracina, K. P., Miyazaki, H., Ishikawa, T., Endo, I., Waters, M. R., Qi, Q., Yan, L., Milstien, S., Spiegel, S., & Takabe, K. (2018). ABCC1-Exported Sphingosine-1-phosphate, Produced by Sphingosine Kinase 1, Shortens Survival of Mice and Patients with Breast Cancer. *Molecular Cancer Research : MCR*, *16*(6), 1059–1070. https://doi.org/10.1158/1541-7786.MCR-17-0353

Zhang, H., Guo, W., Zhang, F., Li, R., Zhou, Y., Shao, F., Feng, X., Tan, F., Wang, J., Gao, S., Gao, Y., & He, J. (2020). Monoacylglycerol Lipase Knockdown Inhibits Cell Proliferation and Metastasis in Lung Adenocarcinoma. *Frontiers in Oncology*, *10*. https://doi.org/10.3389/FONC.2020.559568

Zheng, Y., Wang, L., Zhao, Y., Gong, H., Qi, Y., & Qi, L. (2024). Upregulation of SLAMF8 aggravates ischemia/reperfusion-induced ferroptosis and injury in cardiomyocyte. *International Journal of Cardiology*, *399*. https://doi.org/10.1016/J.IJCARD.2023.131688
